# The Role of N^6^‐Methyladenosine Modification in Health and Disease

**DOI:** 10.1002/mco2.70767

**Published:** 2026-05-18

**Authors:** Linghuan Li, Yuanhai Sun, Wanfang Zheng, Lingqin Li, Yaqian Feng, Minyou Qi, Hanbing Li

**Affiliations:** ^1^ College of Chemistry and Materials Science Zhejiang Normal University Jinhua PR China; ^2^ Institute of Pharmacology Zhejiang University of Technology Hangzhou PR China; ^3^ Taizhou Municipal Hospital Taizhou University Taizhou PR China

**Keywords:** diseases, molecular mechanisms, N^6^‐methyladenosine, therapy

## Abstract

Emerging evidence highlights that N^6^‐methyladenosine (m^6^A), the most prevalent internal RNA modification in eukaryotes, serves as a critical epitranscriptomic regulator of RNA metabolism. This posttranscriptional modification modulates alternative splicing, nuclear export, stability, and translation, thereby regulating various physiological processes. Notably, dysregulation of m^6^A‐associated modifiers (writers/erasers/readers) is implicated in a variety of diseases, such as metabolic disorders and cancer. Despite the rapid progress of m^6^A‐mediated emerging therapeutic strategies, there remains an imperative to bridge the gap between basic epitranscriptomics and clinical application. This review systematically depicts recent advances in understanding m^6^A‐mediated epitranscriptomic regulation, with particular focus on its dual role in maintaining cellular homeostasis and driving disease progression upon dysregulation, provides a dedicated exploration of m^6^A‐regulated mitochondrial remodeling, and outlines cutting‐edge technologies for m^6^A mapping and inhibitors targeting m^6^A modifiers. Furthermore, we conduct an in‐depth exploration of the existing limitations and therapeutic potential associated with targeting m^6^A modification. Acting as a pivotal link between epitranscriptomics and medicine, m^6^A modification provides novel perspectives for developing precision interventions in complex human diseases.

## Graphical abstract

N^6^‐methyladenosine (m^6^A) is the most prevalent internal RNA modification in eukaryotes, acting as a pivotal epitranscriptomic regulator of RNA metabolism. This modification plays a dual role: it maintains physiological homeostasis under normal conditions but drives disease progression when dysregulated. Consequently, elucidating the mechanistic influence of m^6^A on disease pathogenesis may provide novel insights for the development of diagnostics and therapeutics.

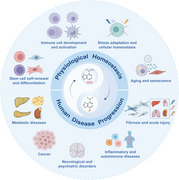

## 1 Introduction

The term “epitranscriptome” was first coined in 2012, emphasizing the crucial role of chemical RNA modifications in regulating its metabolism without altering its nucleotide sequence. Nowadays, over 170 types of posttranscriptional modifications have been sequentially identified, among which N^6^‐methyladenosine (m^6^A) is considered one of the most prevalent modifications in RNA, widely present in various organisms [1]. The m^6^A modification was first discovered in the 1970s, with a frequency of 0.15%–0.6% of all adenosines in mammals [2].

A paradigm shift occurred in the early 2010s with the identification of fat mass and obesity‐associated protein (FTO) and alkB homolog 5 (ALKBH5) as m^6^A‐specific demethylases, challenging the long‐held dogma that RNA modifications are static and irreversible. Concurrently, the discovery of methyltransferase complexes, including methyltransferase like 3 (METTL3)‐METTL14‐Wilms’ tumor 1‐associated protein (WTAP), established the dynamic “writers” of m^6^A, while YTH domain‐containing proteins emerged as evolutionarily conserved “readers” that decode m^6^A marks to regulate RNA splicing, nuclear export, stability, and translation [3]. These breakthroughs, coupled with advances in high‐throughput sequencing technologies such as m^6^A‐sequencing (m^6^A‐seq) and antibody‐based enrichment strategies, catalyzed a renaissance in epitranscriptomics. The historical trajectory of m^6^A research underscores its transformation from a biochemical curiosity to a central mechanism governing diverse biological processes, from development to disease pathogenesis.

Building on the foundational understanding of m^6^A's dynamic regulation, recent research has expanded its scope to unravel the multifaceted roles of epitranscriptomic modifications in bridging RNA biology with organismal physiology and disease. The advent of transcriptome‐wide mapping has revealed that m^6^A deposition is not random but exhibits precise spatiotemporal patterns, fine‐tuning RNA metabolism in response to cellular signals, metabolic states, and environmental stressors. In physiology, m^6^A orchestrates critical processes such as stem cell pluripotency, circadian rhythm regulation, and immune cell differentiation, often through context‐dependent interactions between m^6^A‐modified transcripts and their readers. Conversely, dysregulation of the m^6^A “writers”, “erasers”, or “readers” has been mechanistically linked to pathological states, including cancer progression, metabolic syndromes, cardiovascular pathologies, and autoimmune conditions [4, 5, 6, 7]. For instance, aberrant METTL3‐mediated m^6^A promotes tumorigenesis by enhancing oncogene translation [8], while FTO overexpression in leukemia drives therapeutic resistance by erasing m^6^A‐dependent tumor suppressor signals [9]. Emerging tools, including single‐cell m^6^A sequencing, spatial epitranscriptomics, and CRISPR‐edited methylation switches, are now dissecting how m^6^A heterogeneity within tissues contributes to disease progression or recovery.

Despite the rapid expansion of m^6^A research, the field still faces several important challenges. For example, mechanistic insights, technological advances, and disease‐oriented discoveries are often discussed in isolation rather than integrated together. Therefore, a systematic summary of the molecular basis of m^6^A regulation and its disease relevance is essential to guide future applications. In this review, we summarize the core machinery of m^6^A regulation, together with the main approaches used to profile the m^6^A methylome. We then discuss the physiological functions of m^6^A and its dysregulation across major disease settings, with particular emphasis on metabolic disorders, cancer, neurological and inflammatory diseases, organ injury, and mitochondrial remodeling. Finally, we evaluate current therapeutic strategies targeting the m^6^A axis, including small‐molecule inhibitors and epitranscriptome‐editing approaches, and highlight unresolved questions, translational challenges, and future perspectives. By integrating mechanistic insights with technological and therapeutic advances, this review aims to provide an updated framework for understanding the biological and clinical significance of m^6^A.

## 2 Molecular Machinery and Regulatory Mechanisms of m^6^A Modification

### 2.1 The m^6^A Modification Was Installed by m^6^A Writers

**FIGURE 1:**
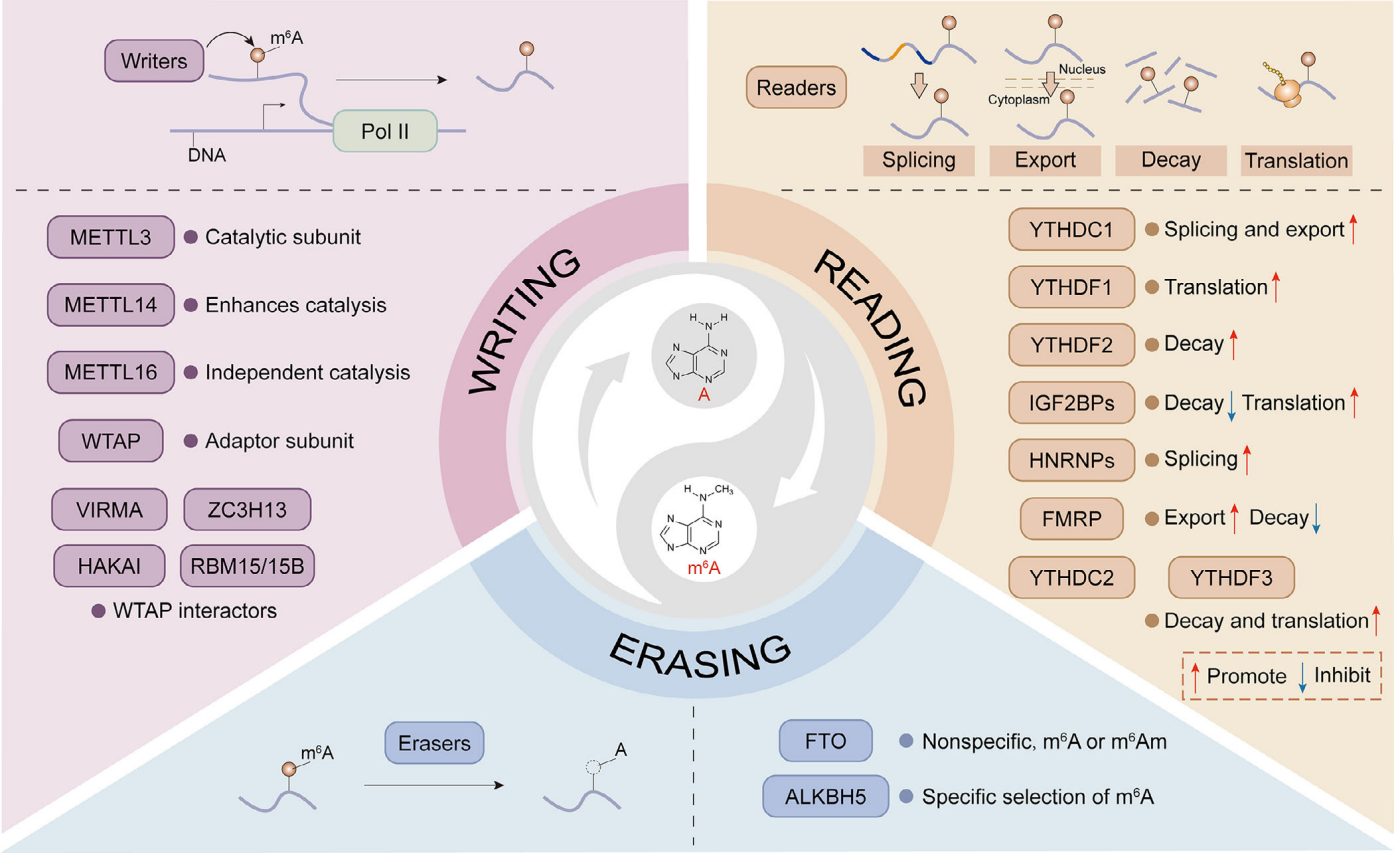
An overview of the RNA metabolic mechanisms by N^6^‐methyladenosine (m^6^A) regulators. m^6^A is installed by the multicomponent methyltransferase complex (MTC) or methyltransferase‐like 16 (METTL16) alone. Within the MTC, methyltransferase‐like 3 (METTL3) and methyltransferase‐like 14 (METTL14) are core components; Wilms’ tumor 1‐associating protein (WTAP) is an adaptor subunit of METTL3, and the components of interaction, including Vir‐like m^6^A methyltransferase‐associated protein (VIRMA), HAKAI, Zinc finger CCCH domain‐containing protein 13 (ZC3H13), and RNA binding motif protein 15/15B (RBM15/15B), assist core components to deposit m^6^A co‐transcriptionally. m^6^A demethylation is removed by either of two m6A demethylases: fat mass and obesity‐associated protein (FTO) and alkB homolog 5 (ALKBH5). m^6^A recognition is done by m^6^A‐binding proteins, including the YT521‐B homology (YTH) domain‐containing protein family (YTHDF1/2/3 and YTHDC1/2), insulin‐like growth factor‐2 mRNA‐binding proteins (IGF2BPs), heterogeneous nuclear ribonucleoproteins (HNRNPs), and fragile X mental retardation protein (FMRP), which mediate splicing, nuclear export, decay, or translation in the nucleus and cytoplasm.

m^6^A is usually installed (Figure 1) by a multicomponent methyltransferase complex (MTC) that contains a catalytic subunit (METTL3) and regulatory subunits (including METTL14, WTAP, VIRMA, HAKAI, ZC3H13, and RBM15/15B) [10]. METTL14 and WTAP, as components that directly bind to METTL3, play a role in enhancing the catalytic activity of METTL3 and recruiting METTL3 to nuclear speckles, respectively. As a regulatory subunit, WTAP‐VIRMA directly interacted with RGG motifs to prevent the binding of dsDNA, thus maintaining the RNA methylation activity of METTL3‐METTL14. Loss of HAKAI destabilized several subunits of MTC, leading to inhibited m^6^A deposition [11]. Furthermore, HAKAI might act as a “bridge” connecting HIZ1 (the plant equivalent of ZC3H13) with core m^6^A writer components [12]. ZC3H13 interacted with RBM15 and WTAP and acted as a linker between these two proteins, thereby stabilizing the MTC and promoting m^6^A deposition on RNA [13]. RBM15/15B bound RNA and recruited MTC to specific sites on RNA [3].

In addition, unlike METTL3/14, METTL16, which is also a member of the methyltransferase family, could independently control the modification of mRNA m^6^A. In the nucleus, METTL16 acted as an m^6^A writer, depositing m^6^A into hundreds of its specific RNA targets [14]. Recently, some METTL family proteins (such as METTL4/7A/7B) that could cause mRNA m^6^A methylation have appeared in our field of view [15, 16, 17]; however, how they participate in the process of m^6^A deposition remains to be explored. Therefore, the types and functions of m^6^A writers are constantly being enriched.

### 2.2 The m^6^A Modification Was Eliminated by m^6^A Erasers

m^6^A can be converted to A by demethylation elimination (Figure 1) by FTO and ALKBH5, which were dependent on Fe^2+^ and α‐ketoglutaric acid [18, 19]. FTO is the first m^6^A “eraser” to be discovered, and its depletion significantly increased the total m^6^A level [19]. Additionally, it is worth mentioning that FTO can remove multiple methyl modifications, including 3‐methyluridine (m^3^U), N1‐methyladenosine (m^1^A), and N^6^, 2’‐O‐dimethyladenosine (m^6^Am) in RNA [20]. Therefore, in view of the fact that the demethylation activity of FTO to mRNA was nonspecific, it is necessary to consider the role of FTO from a comprehensive perspective. ALKBH5 is another m^6^A demethylase that can oxidatively reverse m^6^A in mRNA [18]. Unlike FTO, ALKBH5 has no activity against m^6^Am [21], which might be related to the fact that ALKBH5 does not have a structural fold similar to FTO [22]. Crystallographic and biochemical studies showed that ALKBH5 has a smaller active site cavity than FTO [23]. This allows ALKBH5 to have a more stringent screening mechanism for substrates and cannot accommodate larger modified bases, thus excluding some nonspecific substrates. Additionally, ALKBH5 prefers substrate sequences with the (A/G)m^6^AC motif compared with FTO, consistent with the prevalence of m^6^A in biological contexts, including DRACH motifs [24]. Functionally, ALKBH5 directly converts m^6^A to A without producing an intermediate, but FTO catalyzes this process in two steps [25]. The higher selectivity may be structurally explained by the more hydrophobic residues adjacent to the key HX(D/E) motif in the catalytic pocket of ALKBH5, which diminishes the pocket's affinity for the hydrophilic groups of intermediates like N^6^‐hydroxymethyladenosine and N^6^‐formyladenosine [26]. Therefore, ALKBH5 is highly selective for m^6^A compared with FTO. Furthermore, ALKBH5 is mainly localized in the nucleus, and FTO is localized in both the nucleus and cytoplasm, where it exhibits distinct demethylation preferences [22].

### 2.3 The Fate of m^6^A‐Modified RNAs Was Decoded by m^6^A Readers

m^6^A can be recognized by m^6^A‐binding proteins (Figure 1), which in turn affect the fate of mRNA. m^6^A binding proteins mainly include the YTHDC family (YTHDC1/2), YTHDF family (YTHDF1/2/3), HNRNP family (HNRNPC/G/A2B1), and IGF2BP family (IGF2BP1/2/3). Among them, YTHDC1 promoted the splicing and nuclear export of m^6^A mRNA in the nucleus [27, 28]. YTHDC2 increased the translation efficiency and decreased the mRNA abundance of its targets [29]. YTHDF1 promoted the translation of m^6^A mRNA [30]. YTHDF2 promoted the degradation of m^6^A mRNA by targeting P bodies and recruiting CCR4‐NOT complexes [31, 32]; YTHDF3 and YTHDF1 synergistically promoted the translation of m^6^A mRNA, and cooperated with YTHDF2 to promote the degradation of m^6^A mRNA [33, 34]. HNRNPs were located in the nucleus and mediated m^6^A mRNA splicing [35, 36, 37, 38]. IGF2BPs protected m^6^A mRNA from P‐body degradation and facilitated the translation of mRNA [39]. Fragile X mental retardation protein (FMRP) promoted nuclear export of m^6^A‐modified mRNA [40], and its deficiency could accelerate the degradation of those m^6^A‐marked FMRP targets through YTHDF2, meanwhile leading to the translation of YTHDF1 target transcripts upregulated [41, 42]. Collectively, a number of prospective investigations provided a clear picture of the mechanism of RNA metabolism by m^6^A regulators, but still do not have a comprehensive knowledge of why cells precisely adjust a particular isozyme in response to certain conditions, along with the transcripts that it targets. As well as why some transcripts are methylated at particular periods, and others are not. These common issues need to be fully elucidated.

## 3 Mapping and Quantifying m^6^A: Emerging Tools and Technologies

### 3.1 Transcriptome‐Wide Mapping

**FIGURE 2:**
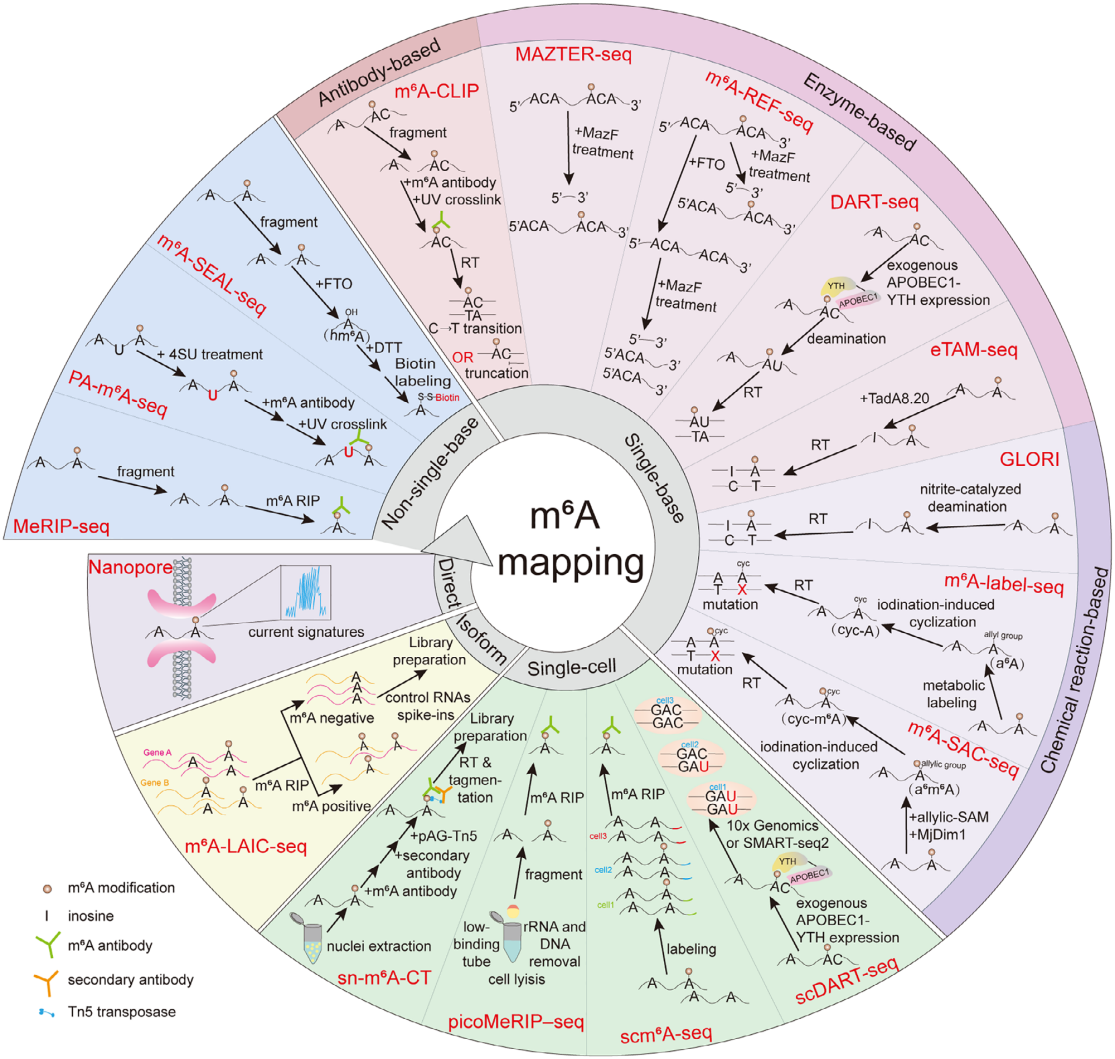
Representative m^6^A mapping strategies classified by detection principle and resolution. Current approaches for transcriptome‐wide m^6^A profiling mainly include nonsingle‐base, single‐base, single‐cell, isoform‐level, and direct RNA detection strategies. Non‐single‐base methods are mainly enrichment‐ or antibody‐based and identify m^6^A‐containing regions without exact nucleotide resolution, whereas single‐base methods achieve site‐specific mapping through antibody‐assisted crosslinking, enzyme sensitivity, or chemical conversion/labeling. Single‐cell approaches extend m^6^A detection to low‐input samples, isoform‐level methods distinguish transcript‐specific methylation patterns, and nanopore sequencing enables direct signal‐based detection of RNA modifications.

As the most prevalent RNA modification, understanding the distribution of m^6^A within RNA holds significant importance for deciphering biological processes. Here, we summarize the development of high‐throughput m^6^A detection technologies (Figure 2).

#### 3.1.1 Nonsingle‐Base Resolution M^6^A Mapping

In 2012, two independent groups first reported a transcriptome‐wide m^6^A mapping approach utilizing m^6^A antibodies, termed methylated RNA immunoprecipitation sequencing (MeRIP‐seq) or m^6^A‐seq [43, 44]. This method entails the isolation and purification of mRNA, followed by fragmentation. Subsequently, m^6^A‐modified fragments are immunoprecipitated using an anti‐m^6^A antibody, and then sequencing is performed. This method is widely used as a foundational technique to determine various biological samples, but it fails to accurately identify specific sites. Otherwise, this method involves cross‐reactivity between the m^6^A antibody and m^6^Am, and requires an input of approximately 150 ng of poly(A) RNA or 500 ng of total RNA, posing significant challenges for the analysis of rare or low‐abundance samples [45, 46].

Photo‐crosslinking‐assisted m^6^A sequencing (PA‐m^6^A‐seq) draws inspiration from photoactivatable ribonucleoside‐enhanced cross‐linking and immunoprecipitation (PAR‐CLIP), offering enhanced resolution of approximately 30 nt [27, 47, 48]. This method involves incubating cells with 4‐thiouridine (4SU), a light‐sensitive ribonucleotide analog, to enable its incorporation into nascent RNA during synthesis. The 4SU‐labeled RNA was induced to undergo covalent cross‐linking with m^6^A antibodies, ultimately leading to a T‐to‐C transition nearby m^6^A. However, this method requires 4SU incubation, rendering it unsuitable for tissue samples. Moreover, its performance is influenced by the bioconversion efficiency of 4SU during incubation, and 4SU treatment may trigger cellular stress responses [47].

In comparison to MeRIP‐seq and PA‐m^6^A‐seq, m^6^A‐SEAL‐seq represents an antibody‐independent approach that employs FTO‐assisted chemical labeling for m^6^A detection. This method utilizes FTO to convert chemically inert m^6^A into a highly reactive intermediate, N^6^‐hydroxymethyladenosine (hm^6^A). Subsequently, m^6^A‐modified RNA enrichment is achieved through addition and thiol‐reactive reactions. This method offers advantages in terms of shorter processing time, reduced costs, and lower RNA input requirements [49].

#### 3.1.2 Single‐Base Resolution M^6^A Mapping

To refine the mapping of m^6^A distribution, researchers employed a multimodal strategy to enhance detection accuracy, incorporating antibody‐based, enzymatic, and chemical approaches.

##### 3.1.2.1 Antibody‐Based Techniques

m^6^A‐CLIP is a methodology analogous to iCLIP, wherein fragmented RNA is incubated with m^6^A antibody, followed by UV‐induced covalent cross‐linking of the antibody to m^6^A‐modified RNA, resulting in the C‐to‐T transition or truncation during reverse transcription [50]. However, this method may yield false‐positive signals owing to the inability of the m^6^A antibody to discriminate m^6^A from m^6^Am or preferential UV cross‐linking to RNA pyrimidine bases, and it lacks single‐nucleotide resolution for distinguishing m^6^A clusters. Thereafter, MeCLIP has simplified the complex [γ‐^32^P] ATP labeling step through 3’RNA adapter ligation to yield high library complexity [51]. Another CLIP‐based method, m^6^ACE‐seq (m^6^A‐cross‐linking‐exonuclease sequencing), eliminates the complicated steps inherent and reduces noise in the previous two approaches [52]. However, these antibody‐based sequencing strategies exhibit limited capacity to discriminate between m^6^A and m^6^Am modifications.

##### 3.1.2.2 Enzyme‐Based Techniques

MAZTER‐seq is an RNase MazF‐dependent method for transcriptome‐wide profiling of m^6^A modifications. MazF enzyme specifically recognizes and cleaves before the ACA sites motifs, but not (m^6^A)CA motifs. Consequently, performing reverse transcription and sequencing on the cleaved RNA enables accurate prediction of m^6^A sites and quantification of m^6^A stoichiometry [53]. As another MazF‐dependent approach, m^6^A‐REF‐seq employs FTO demethylase pretreatment of samples before MazF‐mediated cleavage, thereby ensuring the reliability of m^6^A site identification [54]. Both approaches are well‐suited for analyzing trace samples. Nevertheless, they are constrained by their narrow‐spectrum enzyme substrates targeting the ACA motif, leading to a marked underestimation of m^6^A modification sites compared with actual levels; for example, only 16% of m^6^A modifications are detected in mammalian cells.

Deamination adjacent to RNA modification targets sequencing (DART‐seq) and evolved TadA‐assisted N^6^‐methyladenosine sequencing (eTAM‐seq) have been reported as deaminase‐dependent approaches for single‐base resolution sequencing of m^6^A modifications. Briefly, DART‐seq works by fusing the cytidine deaminase APOBEC1 with the m^6^A‐binding YTH domain. This fusion protein specifically recognizes m^6^A‐modified sites and induces cytosine‐to‐uracil deamination at flanking positions, thereby enabling the mapping of m^6^A modification sites. Although this approach enables mapping of m^6^A sites from low RNA input, transfection efficiency directly influences deamination editing efficacy and restricts its application [55]. Zhu et al. [56] demonstrated that the YTH^D422N^ domain mutation significantly enhanced the recognition efficiency of the fusion protein. Furthermore, eTAM‐seq utilizes the *Escherichia coli*‐derived TadA8.20 deaminase to selectively deaminate unmethylated adenosine to inosine, which is misread as guanine during reverse transcription, thereby enabling transcriptome‐wide mapping and quantitative analysis of m^6^A deposition. Notably, this method achieves quantitative m^6^A detection at specific sites with ultra‐low RNA input (as low as ten cells of RNA), which provides a foundational framework for their potential application in single‐cell m^6^A mapping [57].

##### 3.1.2.3 Chemical Reaction‐Based Techniques

Beyond enzyme‐mediated deamination, the GLORI achieves single‐base m^6^A sequencing through chemical deamination. In brief, this method involves protecting guanosine, followed by nitrite treatment to effectively deaminate unmethylated adenosine into inosine. Subsequently, guanosine is deprotected under alkaline or heated conditions. During reverse transcription, inosine pairs with cytidine and is read as G in sequencing, whereas m^6^A resists deamination [58]. While this method exhibits high reproducibility and cost‐effectiveness, the harsh chemical treatments involved compromise the integrity of RNA. Additionally, this method is unable to distinguish other modifications [58]. Compared with the GLORI, chemical cooperative catalysis‐assisted N^6^‐methyladenosine sequencing (CAM‐seq) operates under mild reaction conditions, thereby minimizing the degradation of RNA. Furthermore, CAM‐seq achieves high‐sensitivity detection with minimal RNA input (as little as 10 ng of mRNA), accompanied by background noise as low as 0.5%, enabling analysis of low‐abundance transcripts [59].

S‐adenosyl methionine analogs, serving as exogenous methyl donors, were incorporated into m^6^A‐label‐seq. This method exploits cellular uptake of Se‐allyl‐L‐selenohomocysteine (allyl‐SeAM)/S‐allyl‐homocysteine (allyl‐SAM) to metabolically label putative m^6^A sites as N^6^‐allyladenosine (a^6^A). Under iodination conditions, a^6^A undergoes cyclization to form N^1^,N^6^‐cyclized adenosine (cyc‐A). The cyc‐A‐induced misincorporation at the opposite site in cDNA during reverse transcription indicates the m^6^A sites. Compared with DART‐seq, m^6^A‐REF‐seq, and m^6^A‐CLIP, m^6^A‐label‐seq exhibits superior sensitivity for detecting clustered m^6^A sites [60, 61]. Additionally, by applying a selenium‐based cosubstrate analog to install a propargyl group at m^6^A sites, Hartstock et al. established a method to profile mRNA m^6^A methylome [62, 63]. Inspired by this approach, Mikutis et al. [64] developed a substrate‐hijacking and RNA degradation strategy for methylation detection. These methods require cellular uptake of SAM analogs, thereby rendering both labeling efficiency and sampling time critical for the identification of m^6^A sites. Furthermore, treatment with SAM analogs may induce cellular stress responses.

To overcome limitations inherent to cellular incubation with SAM analogs, m^6^A‐SAC‐seq employs MjDim1, a dimethyltransferase derived from *Methanocaldococcus jannaschii* [65], that selectively transfers an allyl group to m^6^A, resulting in its conversion to N^6^‐allyl,N^6^‐methyladenosine (a^6^m^6^A) in the presence of allylic‐SAM. Subsequent treatment with iodine induces cyclization of a^6^m^6^A, yielding cyclized a^6^m^6^A, which will be read as a mutation. Thus, the position and quantitation of m^6^A deposition can be profiled through high‐throughput sequencing. Although this approach requires only approximately 30 ng of input RNA, MjDim1 exhibits a motif preference of GAC over AAC, potentially compromising the labeling of all m^6^A sites [66].

Additionally, chemical approaches independent of deamination and SAM‐mimetic labeling have been developed, such as 4‐position selenium‐modified deoxythymidine triphosphates (4SedTTP)‐involved and FTO‐assisted strategy, as well as m^6^A‐ORL‐seq. The 4SedTTP‐involved and FTO‐assisted strategy employs 4SedTTP to inhibit its own base‐pairing with m^6^A, generating specific reverse‐transcription truncation signals that induce premature termination of m^6^A‐containing transcripts; meanwhile, RNA samples treated with the m^6^A demethylase FTO serve as controls [67]. The m^6^A‐ORL‐seq method adopts a selective chemical labeling method for single‐base m^6^A detection. This method permits detection of low‐abundance methylated samples, but it risks inducing deamination of nontarget bases and exhibits sensitivity to reaction inefficiencies, potentially yielding false‐positive outcomes [68].

### 3.2 Single‐Cell and Isoform‐Specific Approaches

Traditional bulk‐cell methylation sequencing masks cell‐type‐specific dynamic methylation changes, impeding insights into epitranscriptomic heterogeneity across diverse cellular populations. Recent advances in single‐cell m^6^A sequencing technologies (e.g., scDART‐seq, scm^6^A‐seq, and sn‐m^6^A‐CT) have heralded a transformative era for resolving cell‐type‐resolved m^6^A modifications and their functional roles.

By integrating droplet‐based (10x Genomics) scRNA‐seq into DART‐seq, scDART‐seq realizes the discrimination of methylation signatures among cellular subpopulations and represents the first m^6^A detecting method at the single‐cell level [69, 70]. The scDART‐seq enables the detection of variations in the distribution and abundance of m^6^A sites across individual cells, permitting the discrimination of cellular subpopulations based on their RNA methylation signatures independently of gene expression fluctuations. However, scDART‐seq requires transfection of the APOBEC1‐YTH plasmid into cells, which restricts its application, particularly in some primary cells and in in vivo experiments.

Single‐cell m^6^A sequencing (scm^6^A‐seq) is a technique developed through RNA multiplex labeling and MeRIP‐seq, which can simultaneously profile the m^6^A methylome and transcriptome in single cells. Yao et al. utilized this approach to uncover m^6^A‐dependent epitranscriptomic asymmetry between blastomeres of a two‐cell embryo during early development and identified multiple transcription factors with differential m^6^A modifications [71].

PicoMeRIP‐seq is another m^6^A antibody‐dependent single‐cell m^6^A mapping technique that does not require RNA labeling. Li et al. achieved transcriptome‐wide m^6^A sequencing of picogram poly(A) RNA and as few as 10 cells through optimizing sample recovery and signal‐to‐noise ratio. In this method, several key steps were improved as follows: (1) increasing the concentrations of sodium dodecyl sulfate and sodium chloride, and performing vigorous vortexing instead of gentle mixing; (2) using low‐binding tubes; (3) employing commercially available m^6^A antibodies, etc. picoMeRIP‐seq has revealed the association between the profile of m^6^A regulatory mechanisms and fertility as well as developmental defects through m^6^A mapping in single oocytes and embryos [72]. In addition, sn‐m^6^A‐CT was developed to simultaneously profile the transcriptomes and methylomes in thousands of single nuclei. Briefly, the procedure labels m^6^A‐RNA in isolated nuclei with an m^6^A antibody and then uses a secondary antibody and Tn5 transposase to bind RNA‐antibody complexes. Following this, adapter‐tagged RNA/cDNA hybrids are obtained through reverse transcription and tagmentation. However, sn‐m^6^A‐CT is an antibody‐based technique that suffers from low resolution, m^6^Am cross‐reactivity, and lacks quantitative stoichiometric information for individual m^6^A sites [73].

As mentioned above, the majority of existing methods involve fragmentation and potential degradation, which compromises the integrity of isoform characterization. Importantly, detecting m^6^A modification levels in alternative RNA isoforms holds significant meaning, considering their crucial role in regulating transcript expression [74]. m^6^A‐LAIC‐seq was developed to determine differences in m^6^A modification levels and site‐specific patterns among individual transcripts of each gene. In this method, full‐length transcripts are immunoprecipitated with an anti‐m^6^A antibody, then reverse‐transcribed and sequenced. This approach allows for the detection of differential isoform usage in methylated and nonmethylated transcripts of individual genes [75, 76]. However, this method quantifies the m^6^A stoichiometry on the isoform rather than the individual m^6^A site.

Overall, all the sequencing methods mentioned above require cDNA synthesis and amplification, which may lead to base mismatches and deletions. This limitation has spurred the development of approaches for the direct detection of RNA modifications. In 2018, Oxford Nanopore Technologies Ltd developed nanopore sequencing technology that enables direct long‐read RNA sequencing [77]. The technology employs transmembrane nanopore proteins as biosensors to capture subtle ionic current fluctuations induced by single‐stranded DNA/RNA translocation through the nanopore [78]. Advancements in Oxford Nanopore sequencing technology have facilitated the concurrent detection of multiple RNA modifications [79, 80]. Computer systems for signal discrimination are being continuously optimized to improve detection efficiency and accuracy, such as DRUMMER [81], ELIGOS [82], and JACUSA2 [83], which are based on algorithms for base‐calling error rates; as well as MINES [84], xPore [85], DENA [86], CHEUI [87], and Nanocompore [88], which rely on raw ionic current signals. In addition, nanopore direct RNA sequencing coupled with DART can accurately identify and quantify m^6^A modifications across RNA isoforms, the intricate dynamics and regulatory complexities of these modifications [89]. As an emerging technology, numerous challenges remain to be addressed, such as sample degradation during storage, particularly for mRNA, and the accuracy of this technique requires further optimization.

## 4 Physiological Roles of m^6^A in Cellular and Organismal Homeostasis

**FIGURE 3:**
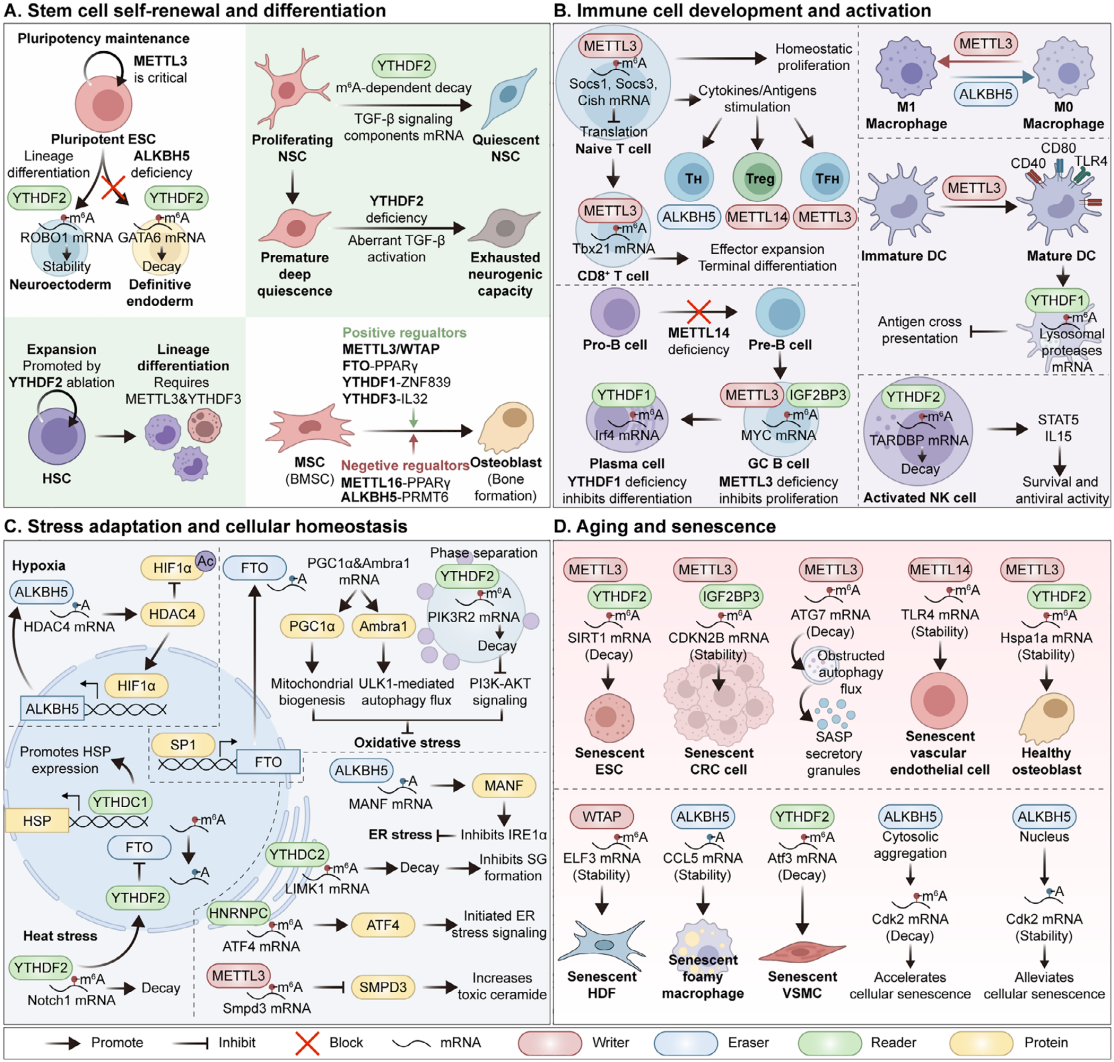
N^6^‐methyladenosine (m^6^A) modification affects a wide range of physiological processes. (A) In stem cells, m^6^A modifiers regulate embryonic stem cell (ESC) pluripotency maintenance and lineage differentiation, neural stem cell (NSC) quiescence, hematopoietic stem cell (HSC) expansion and differentiation, and mesenchymal stem cell (MSC) osteogenic differentiation. (B) In the immune system, m^6^A modifiers regulate T cell homeostasis and differentiation, macrophage polarization, dendritic cell (DC) maturation, B cell development, and natural killer (NK) cell survival and antiviral activity. (C) m^6^A modifiers dynamically reshape RNA fate (stability, nuclear export, and translation) under conditions of hypoxia, oxidative stress, heat shock, and endoplasmic reticulum (ER) stress. (D) During aging and senescence, m^6^A‐dependent pathways influence autophagy flux, senescence‐associated secretory phenotype (SASP) secretion, inflammatory signaling, and senescence‐related phenotypes in multiple cell types.

The m^6^A modification is not merely a structural alteration of RNA but a dynamic regulatory mechanism that profoundly influences a wide array of physiological processes (Figure 3). Its roles extend from controlling the fundamental fate of RNA molecules to governing complex organismal functions, highlighting its importance in maintaining cellular and systemic homeostasis.

### 4.1 Stem Cell Self‐Renewal and Differentiation

Stem cells are fundamental to the development of multicellular organisms, tissue regeneration, and maintenance of physiological homeostasis. Their unique biological identity is defined by two properties: first, the capacity of self‐renewal, whereby cell division yields progeny that retain the parental phenotype to sustain the stem cell pool; second, multipotency, the ability to differentiate into diverse functional lineages upon exposure to specific inductive signals. While transcription factor networks and chromatin remodeling have long been recognized as the primary regulatory mechanisms of these processes, the emergence of epitranscriptomics has revealed that gradually realized that chemical modifications at the RNA level, especially m^6^A, serve as indispensable “molecular switches” in determining stem cell fate (Figure 3A).

The dynamic regulation of m^6^A is essential for the cell‐fate transitions of embryonic stem cells (ESCs) [90]. m^6^A modification, catalyzed by the methyltransferase METTL3, regulates pluripotency maintenance and lineage differentiation [91]. Mechanistically, HDAC2 recruits METTL3 to mediate m^6^A deposition on target genes and subsequently regulates RNA stability and translation through IGF2BPs and YTHDC2. Similarly, the m^6^A reader YTHDF2 is important for human embryonic stem cells (hESCs) differentiation, especially toward the ectoderm, but remains dispensable for pluripotency maintenance [92]. Evidence suggests that m^6^A‐modified *ROBO1* (roundabout guidance receptor 1) mRNA is a potential target of YTHDF2 during neuroectodermal specification. Furthermore, deletion of the m^6^A demethylase ALKBH5 severely impairs definitive endoderm differentiation, as ALKBH5^−/−^ hESCs fail to navigate the primitive streak transition. This defect is driven by m^6^A hypermethylation of the 3′‐untranslated region (3′UTR) of *GATA6* transcripts, which destabilizes *GATA6* mRNA in a YTHDF2‐dependent manner [93].

During neurogenesis, neural stem cells (NSCs) must either exit the cell cycle to differentiate into neurons and neuroglia or enter a quiescent state to maintain the adult stem cell reservoir. YTHDF2 constrains the expression of TGF‐β signaling components by mediating m^6^A‐dependent mRNA decay [94]. Consequently, YTHDF2 deficiency leads to the aberrant activation of TGF‐β signaling, forcing proliferating hippocampal NSCs to prematurely exit the cell cycle and enter deep quiescence, which ultimately exhausts neurogenic capacity.

Recent studies have further elucidated the role of m^6^A in hematopoietic stem cells, where the loss of modifiers such as METTL3 or YTHDF3 impairs self‐renewal and lineage differentiation [95, 96, 97, 98]. Conversely, the ablation of YTHDF2 promotes hematopoietic stem cell expansion and regeneration by preventing the clearance of m^6^A‐modified transcripts essential for stemness [99, 100].

m^6^A modification precisely orchestrates the osteogenic differentiation of mesenchymal stem cells (MSCs), especially bone marrow mesenchymal stem cells (BMSCs). While METTL3 and WTAP function as positive regulators of osteogenic differentiation in BMSCs, METTL16 suppresses this process [101, 102, 103]. Specifically, METTL16 enhances PPARγ transcription, which triggers ferroptosis and inhibits osteogenic differentiation. In contrast, the m^6^A demethylase FTO promotes osteogenic differentiation by reducing *PPARγ* mRNA stability via an m^6^A‐YTHDF1‐dependent manner [104]. Interestingly, another demethylase, ALKBH5, serves as a negative regulator of osteogenic differentiation by accelerating the degradation of *PRMT6* (protein arginine methyltransferase 6) mRNA, thereby inhibiting PI3K/AKT signaling [105]. The involvement of readers is equally critical: YTHDF1 and YTHDF3 both facilitate osteogenic differentiation of BMSCs, albeit through distinct mechanisms; for instance, YTHDF1 enhances *ZNF839* (zinc finger protein 839) translation, whereas YTHDF3 stabilizes *IL32* (interleukin 32) mRNA [106, 107].

### 4.2 Immune Cell Development and Activation

m^6^A modification serves as a pivotal determinant of immune cell fate and functional plasticity (Figure 3B). This section provides a systematic overview of the physiological roles of m^6^A in the development and differentiation of lymphocyte (T and B cells), as well as the activation and effector functions of innate immune cells, including macrophages, dendritic cells (DCs), and natural killer (NK) cells.

The T cell life cycle is intricately regulated by m^6^A modification. Naive T cells (antigen not encountered) rely on METTL3‐mediated m^6^A modification to suppress the activity of the suppressor of cytokine signaling (SOCS) family. This suppression facilitates the activation of interleukin‐7 (IL‐7) signaling, thereby maintaining cellular survival and homeostatic proliferation at basal levels [108]. Upon stimulation by cytokines or antigens, CD4^+^ naive T cells differentiate into various T helper (T_H_) effector subsets (T_H_1, T_H_2, T_H_17) or regulatory T cells (Tregs) to orchestrate specific immune responses. Notably, ALKBH5 expression is significantly upregulated in T_H_1, T_H_2, and Tregs compared with naive T cells, suggesting its role in lineage‐specific pathogenicity [109]. Furthermore, METTL14 deficiency impairs the induction of naive T cells into Tregs [110], while METTL3 is necessary for the expression of canonical T follicular helper (T_FH_) cell signature genes like *Tcf7*, thereby promoting T_FH_ differentiation [111]. Beyond CD4^+^ lineage commitment, m^6^A modification also regulates the effector differentiation of CD8^+^ T cells; specifically, METTL3 stabilizes *Tbx21* (T‐box transcription factor 21) mRNA stability to sustain effector expansion and terminal differentiation, which are critical for memory formation and secondary immune recall [112].

In B cells, m^6^A modification is essential for early development, activation, and rapid proliferation. Ablation of *Mettl14* results in a severe developmental blockage at the Pro‐B to Pre‐B and the large‐to‐small Pre‐B transitions, a phenotype largely attributed to impaired IL‐7 receptor signaling [113]. Within germinal centers, B cells undergo somatic hypermutation and affinity maturation [114]. These germinal center B cells (centroblasts) exhibit high proliferation rates driven by the proto‐oncogene *MYC* [115]. Although *MYC* mRNA is generally unstable, METTL3 and IGF2BP3 maintain the cell cycle, oxidative phosphorylation, and the overall germinal centers reaction by stabilizing *MYC* mRNA [116]. In the final stages of B cell differentiation, the reader YTHDF1 enhances the stability of *Irf4* (interferon regulatory factor 4) mRNA, facilitating the transition into antibody‐secreting plasma cells [117].

In the context of innate immunity, m^6^A modification acts as a molecular switch for macrophage polarization. METTL3 and ALKBH5 dynamically regulate macrophage M1 polarization [118]. *Mettl3* deficiency inhibits imiquimod‐induced M1 polarization, while *Alkbh5* deficiency promotes it. In DCs, which are responsible for antigen uptake and presentation, METTL3 is required for activation and maturation by promoting the translation of costimulatory molecules (CD40, CD80) and Toll‐like receptor 4 (TLR4) signaling molecules [119]. Additionally, m^6^A plays an unexpected role in regulating DCs‐mediated antigen cross‐presentation. In *Ythdf1*‐deficient DCs, the translational efficiency of lysosomal proteases is reduced, which preserves antigen integrity and enhances the ability of DCs to cross‐present antigens to CD8^+^ T cells, ultimately eliciting a more potent antitumor response [120]. The survival and antiviral activity of NK cells depend on YTHDF2. In activated NK cells, YTHDF2 maintains STAT5 signaling by degrading *TARDBP* (TAR DNA binding protein) mRNA, forming a positive feedback loop that sustains IL‐5 responsiveness [121].

The maturation of single‐cell epitranscriptomic sequencing will enable a more granular analysis of m^6^A heterogeneity across immune subpopulations. Current evidence strongly suggests that pharmacological modulation of m^6^A modifiers (such as METTL3 inhibitors) represents a promising therapeutic strategy for managing autoimmune diseases and enhancing the efficacy of cancer immunotherapy.

### 4.3 Stress Adaptation and Cellular Homeostasis

The capacity of cells and organisms to maintain homeostasis in the face of environmental fluctuations, metabolic challenges, and pathologies is fundamental to biological survival. m^6^A modification represents a critical regulatory layer in this process, remodeling the cellular proteome by dynamically reshaping RNA fate (stability, nuclear export, and translation) under conditions of hypoxia, oxidative stress, heat shock, and endoplasmic reticulum stress (Figure 3C).

Central to the hypoxic response are hypoxia‐inducible factors (HIFs), the primary transcriptional drivers of metabolic reprogramming. In this context, m^6^A modification constitutes a key regulator of the HIF signaling axis. Under hypoxic conditions, ALKBH5 removes m^6^A marks from histone deacetylase type 4 (HDAC4) transcripts, resulting in an accumulation of *HDAC4* mRNA and protein [122]. Elevated HDAC4 subsequently deacetylates hypoxia inducible factor 1 alpha (HIF1α), which protects the latter against ubiquitin‐mediated degradation and enhances its stability. This stabilization establishes a potent positive feedback loop, as stabilized HIF1α further transactivates *ALKBH5* expression.

Oxidative stress, arising from an imbalance in reactive oxygen species (ROS) homeostasis, is similarly modulated by epitranscriptomic changes. FTO is essential for maintaining mitochondrial integrity and limiting ROS generation. In cardiomyocytes, FTO overexpression stabilizes Peroxisome proliferator‐activated receptor (PPAR)‐gamma coactivator‐1α (PGC1α) transcripts, thereby upregulating mitochondrial markers such as superoxide dismutase (SOD2), mitochondrial transcription factor A (TFAM), and cytochrome c oxidase I (COXI) [123]. This pathway is further influenced by the transcription factor SP1, which activates the transcription of FTO. The resulting m^6^A demethylation of *Ambra1* mRNA enhances its stability, triggering ULK1 (unc‐51 like autophagy activating kinase 1)‐mediated autophagy flux to inhibit oxidative stress [124]. In addition to endogenous stressors, exogenous toxins (such as arsenite) can also induce a distinct stress response characterized by YTHDF2 phase separation. In human keratinocytes, arsenite‐induced YTHDF2 liquid–liquid phase separation facilitates the degradation of *PIK3R2* (phosphoinositide‐3‐kinase regulatory subunit 2) mRNA, which suppresses PI3K‐AKT signaling and exacerbates oxidative stress [125].

Heat stress (HS) induces a cellular response leading to profound reprogramming of the transcriptome. YTHDC1 binds to m^6^A‐modified heat shock protein (HSP) transcripts to promote their expression [126]. Interestingly, under HS, the YTHDF2 protein, which is normally located in the cytoplasm and promotes *Notch1* mRNA decay, translocates into the nucleus, thereby resulting in reduced *Notch1* mRNA decay [127]. Moreover, studies have demonstrated that m^6^A is preferentially deposited into the 5’UTR of newly transcribed mRNA in response to HS, which results from YTHDF2 nuclear translocation [128]. Nuclear YTHDF2 also protects the m^6^A modification of the 5′UTR from removal by the demethylase FTO. These findings indicate that YTHDF2 does not simply act as an m^6^A reader for mRNA decay but also exhibits its protective function under heat stress.

m^6^A orchestrates the unfolded protein response (UPR) within the endoplasmic reticulum (ER) to maintain proteostasis. In renal cell carcinoma, deletion of von Hippel–Lindau (VHL) triggers chronic ER stress. Under such conditions, MANF (mesencephalic astrocyte‐derived neurotrophic factor) is upregulated via an ALKBH5‐mediated m^6^A modification, which in turn exerts a cytoprotective effect by binding to phosphorylated inositol‐requiring enzyme‐1α and inhibiting its activation [129]. Similarly, YTHDC2 alleviates ER stress by destabilizing *LIMK1* (LIM domain kinase 1) mRNA, thereby inhibiting stress granule (SG) formation [130]. Other readers, such as HNRNPC, may exacerbate stress by promoting the translation of activating transcription factor 4 (ATF4) [131]. METTL3 and METTL14 also play a key role in maintaining ER homeostasis [132, 133]. Loss of METTL3 increased SMPD3 (sphingomyelin phosphodiesterase 3) expression, which in turn results in sphingolipid metabolism rewiring, leading to mitochondrial damage and persistent ER stress [134].

### 4.4 Aging and Senescence

Dysregulated m^6^A modification is one of the core mechanisms driving cellular senescence and organic aging. It is critical to clarify how m^6^A modification affects cellular senescence through molecular mechanisms (Figure 3D). The METTL3/YTHDF2 axis accelerates ESCs’ senescence by facilitating the targeted degradation of sirtuin 1 (SIRT1) mRNA [135]. A similar pro‐senescence role is observed in colorectal cancer cells via the METTL3/IGF2BP3/CDKN2B (cyclin‐dependent kinase inhibitor 2B) regulatory axis [136]. Conversely, METTL3 displays antagonistic effects toward aging in other lineages; it inhibits the senescence of osteoblasts by stabilizing *Hspa1a* (heat shock protein 1A) mRNA in a YTHDF2‐dependent manner [137]. Given its dual role in promoting osteogenic differentiation and inhibiting senescence, METTL3 represents a high‐priority therapeutic target for osteoporosis. Furthermore, m^6^A serves as a critical link between impaired proteostasis and aging. METTL3 suppresses ATG7 (autophagy‐related 7) expression by increasing the m^6^A modification on its transcripts, thereby obstructing autophagic flux and eliciting the senescence‐associated secretory phenotype (SASP), which ultimately drives osteoarthritis progression [138]. Furthermore, the upstream regulation of METTL3 itself is equally vital to the aging process. The E3 ubiquitin ligase PRKN (Parkin) has been identified as a senescence‐associated regulator that facilitates the K48‐linked polyubiquitination and subsequent proteasomal degradation of METTL3 at Lys164 [139]. This PRKN‐mediated downregulation of METTL3 exacerbates telomere dysfunction and accelerates cellular decline.

Other components of the methyltransferase complex, including METTL14 and WTAP, also contribute to the cellular senescence. METTL14 promotes cellular senescence in vascular endothelial cells by enhancing the stability of *TLR4* mRNA [140], while WTAP drives the senescence of human dermal fibroblasts by upregulating the ELF3 (E74‐like ETS transcription factor 3)/IRF8 axis [141]. In addition to methyltransferases, demethylase ALKBH5 also plays a key role in cellular senescence. The cytosolic aggregation of ALKBH5 has been shown to accelerate senescence by promoting m^6^A hypermethylation of *Cdk2* (cyclin‐dependent kinase 2) mRNA; remarkably, this process is reversible, as restoring the nuclear translocation of ALKBH5 (either through NLS‐tagging or administration of m^6^A‐labeled RNA) alleviates the senescent phenotype [142]. Additionally, ALKBH5 promoted senescent foamy macrophage formation through the CCL5 (CC chemokine ligand 5)/CCR5 (CC chemokine receptor 5)/autophagy signaling pathway [143]. Furthermore, the m^6^A “reader” YTHDF2 has been implicated in vascular smooth muscle cell senescence through its ability to destabilize *Atf3* mRNA [144].

Taken together, m^6^A modification has transitioned from a basic regulator of RNA metabolism to a key epitranscriptomic driver of aging. It promotes or delays aging in cells through its tissue‐specific molecular regulation, and its imbalance directly leads to pathological changes such as atherosclerosis, inflammation, and cancer.

## 5 m^6^A Dysregulation and Disease Mechanisms

**FIGURE 4:**
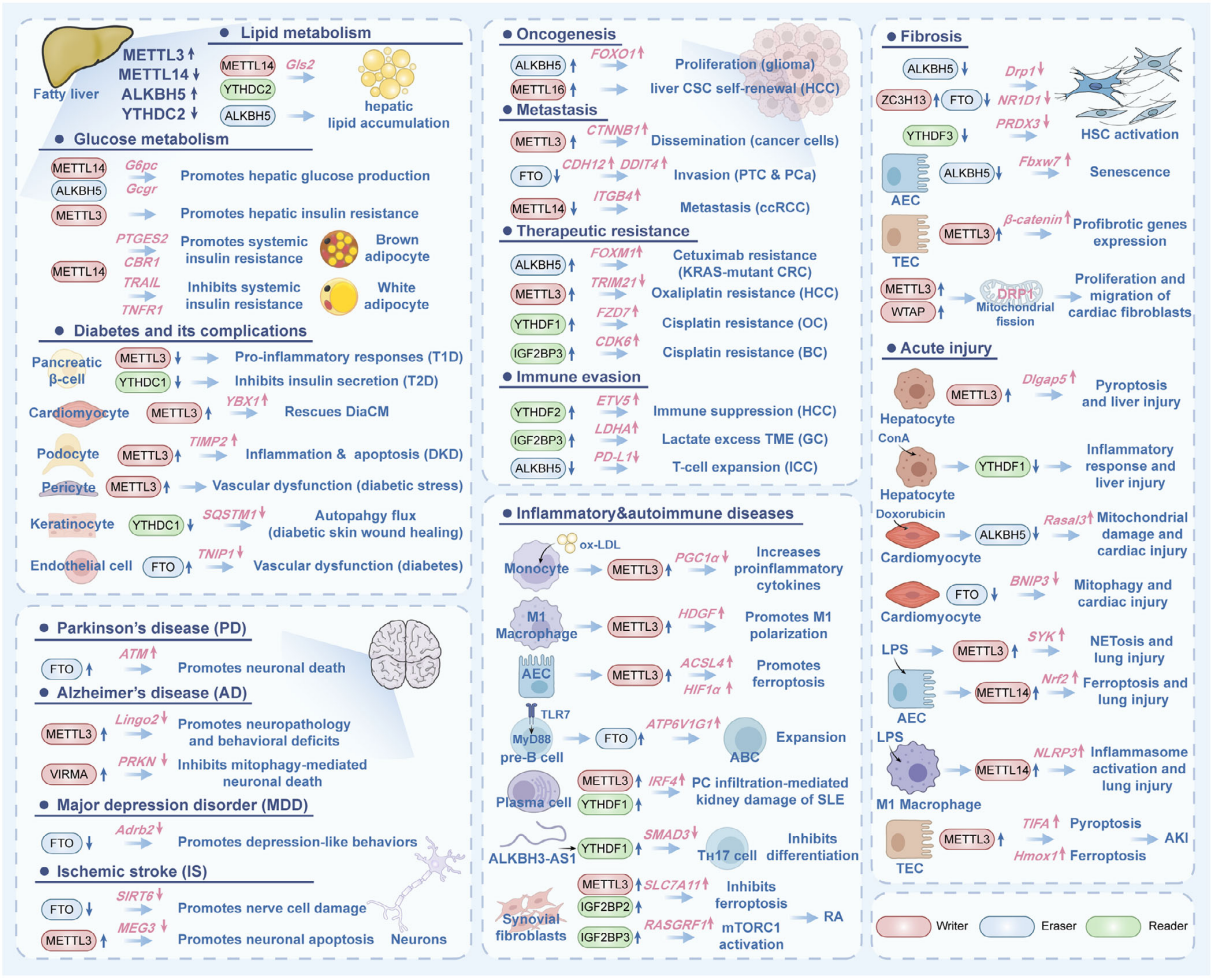
The multifaceted roles of N^6^‐methyladenosine (m^6^A)‐mediated epitranscriptomic regulation in human diseases. m^6^A modifiers‐mediated pathways link altered RNA m^6^A regulation to fatty liver disease, glucose and diabetes‐related complications, cancer progression and therapy resistance, neurological diseases, inflammatory and autoimmune disorders, fibrosis, and acute tissue injury. In each panel, the dysregulated m^6^A modifiers, their key downstream targets, and the resulting biological effects are indicated to highlight how distinct m^6^A axis drive disease‐specific phenotypes.

To date, m^6^A modification has been shown to affect various processes, while the dysregulation of m^6^A modification is associated with various diseases. This part systematically elucidates the multifaceted roles of m^6^A‐mediated epitranscriptomic regulation in metabolic diseases, cancer, neurological and psychiatric disorders, inflammatory and autoimmune diseases, fibrosis, and acute injury, dissecting its molecular mechanisms across pathological dysregulation (Figure 4).

### 5.1 m^6^A‐Regulated in Metabolic Diseases

For the last few decades, the escalating pandemic of metabolic disorders, particularly obesity, metabolic dysfunction‐associated fatty liver, and diabetes, has emerged as a critical threat to global public health. Converging evidence from multi‐institutional cohorts has established that m^6^A modification dynamically modulates the pathogenesis of metabolic diseases [145, 146].

#### 5.1.1 m^6^A Regulates Glucose and Lipid Metabolism in Obesity and Fatty Liver

In the context of metabolic dysfunction‐associated fatty liver disease (MAFLD), the expression of METTL14 is significantly reduced in the livers of both human patients and murine models [147]. Hepatocyte‐specific knockout of Mettl14 exacerbates hepatic lipid deposition, injury, and fibrosis, whereas its overexpression mitigates these pathological features in high‐fat diet (HFD)‐fed mice. Mechanistically, METTL14 deletion reduces the translational efficiency of *GLS2* mRNA via YTHDF1 through m^6^A modification, resulting in increased oxidative stress, which subsequently recruits inflammatory monocyte‐derived macrophages to drive disease progression through the MyD88/NF‐κB pathway. Conversely, the METTL14‐METTL3 complex is upregulated in the liver under obesity, promoting gluconeogenesis through m^6^A modification of *G6pc* mRNA, with YTHDF1/3 involved in this process [148]. Consistently, METTL3 levels are elevated in the liver under HFD conditions, which exacerbates liver metabolic dysfunction and insulin resistance [149]. Additionally, the demethylase ALKBH5 was downregulated in fatty liver compared with normal liver in mice [150]. Interestingly, another study showed that ALKBH5 is upregulated and activated in the liver under obese conditions, promoting abnormal glycolipid metabolism via the GCGR and mTORC1 signaling pathway. Knocking down *Alkbh5* in hepatocytes significantly improves glucose and lipid homeostasis, suggesting its potential as a therapeutic target [151]. This may be related to the time of establishing the fatty liver model in mice and the formula of the high‐fat diet, which needs to be further investigated. On the other hand, the m^6^A “reader” YTHDC2 is downregulated in fatty liver. Its deletion promotes the stability of lipogenesis‐related genes and increases lipid deposition, while overexpression ameliorates steatosis and insulin resistance by destabilizing lipogenic transcripts, including *Srebp1c*, *Fasn*, *Scd1*, and *Acc1* [152].

In adipose tissue, METTL14‐mediated m^6^A methylation inhibits β‐adrenergic signaling and lipolysis. Adipocyte‐specific knockout of *Mettl14* enhances lipolysis and protects against HFD‐induced obesity, insulin resistance, and MAFLD [153]. Additionally, METTL14 regulates brown adipocyte function by promoting YTHDF2/3‐dependent decay of *Ptges2* and *Cbr1* mRNAs, thereby impairing prostaglandin biosynthesis and systemic insulin sensitivity, independently of UCP1 [154]. Conversely, *Mettl14*‐knockout in WAT induces adipocyte apoptosis and systemic insulin resistance [155].

#### 5.1.2 m^6^A in Diabetes and Its Complications

In pancreatic β‐cells, METTL3 expression fluctuates dynamically during type 1 diabetes, characterized by an initial increase followed by a rapid decline; its downregulation promotes pro‐inflammatory innate immune responses [156]. Conversely, in dilated cardiomyopathy (DiaCM), cardiac METTL3 expression and m^6^A modification are downregulated, a phenomenon exacerbated by diabetes. Exercise has been shown to rescue DiaCM by upregulating METTL3, which enhances Nrf2 signaling via m^6^A‐dependent stabilization of YBX1 (Y‐box binding protein 1) [157]. In contrast to its role in the heart, METTL3 is overexpressed in podocytes in diabetic kidney disease, where it exacerbates inflammation and apoptosis through IGF2BP2‐mediated m^6^A modification of *TIMP2* (tissue inhibitor of metalloproteinase 2) mRNA and consequent activation of the Notch pathway [158]. Similarly, METTL3 is significantly upregulated in retinal pericytes under diabetic stress, and m^6^A accumulation promotes vascular dysfunction by facilitating YTHDF2‐mediated decay of pericyte marker transcripts [159].

Beyond METTL3, other m^6^A regulators contribute to diabetic pathogenesis. Lipotoxicity‐induced downregulation of the nuclear reader YTHDC1 impairs insulin secretion and compromises β‐cell identity in type 2 diabetes [160]. In diabetic keratinocytes, hyperglycemia suppresses YTHDC1, impairing autophagic flux and wound healing by destabilizing *SQSTM1* (sequestosome 1) mRNA [161]. Furthermore, endothelial‐specific FTO deficiency paradoxically mitigates diabetes‐induced vascular dysfunction by erasing m^6^A marks on *TNIP1* (TNFAIP3 interacting protein 1) mRNA, thereby increasing its expression and suppressing inflammatory signaling [162].

### 5.2 m^6^A‐Regulated in Cancer

#### 5.2.1 Oncogenic m^6^A Rewiring of mRNA Stability

Recent studies have shown that abnormal methylation of m^6^A is often associated with tumorigenesis and development. m^6^A modification is closely related to malignant transformation and tumorigenesis. Studies have shown that ALKBH5 exhibits significant upregulation in glioma, where it facilitates tumor progression by demethylating m^6^A modifications on forkhead box O1 (FOXO1) mRNA. This process results in the destabilization of *FOXO1* mRNA in a YTHDC1‐dependent manner, thereby enhancing malignant phenotypes [163]. Additionally, METTL16 is overexpressed in hepatocellular carcinoma (HCC), particularly in cancer stem cells (CSCs), where it promotes self‐renewal and tumorigenesis through both m^6^A‐dependent and m^6^A‐independent mechanisms [164]. Notably, METTL16 facilitates ribosomal biogenesis and augments the translation of oncogenic factors such as eIF3a, thereby forming a pro‐tumorigenic axis that is critical for HCC maintenance [14]. While both modifiers converge on posttranscriptional regulation, ALKBH5 predominantly destabilizes tumor suppressors, whereas METTL16 enhances translation efficiency and ribosome function, highlighting context‐specific rewiring of m^6^A‐mediated RNA metabolism. Interestingly, the methyltransferase domain of METTL16 represents a promising therapeutic target, emphasizing the potential for inhibiting both its catalytic and scaffolding functions. These findings suggest that, despite shared associations with poor prognosis, the functional outcomes of m^6^A modifiers are highly tissue‐specific, necessitating tailored therapeutic strategies.

The role of m^6^A in epithelial‐mesenchymal transition (EMT) and metastasis further illustrates its tissue‐specific nature. Studies have demonstrated that β‐catenin is critical for METTL3‐regulated dissemination of cancer cells. For example, METTL3 regulates the dissemination of cancer cells through dual β‐catenin‐dependent mechanisms: it modulates the stability of *CTNNB1* (catenin beta 1) mRNA and influences its membrane localization via c‐Met expression [165]. Similarly, METTL3‐mediated m^6^A modification stabilizes Lnc‐TSPAN12 in HCC, which scaffolds the SENP1‐EIF3I complex to activate Wnt/β‐catenin signaling and promote metastasis [166]. In clear cell renal cell carcinoma, however, METTL14 acts as a metastasis suppressor by depositing m^6^A marks on integrin β‐4 (ITGB4) mRNA, leading to YTHDF2‐mediated decay. Downregulation of METTL14 elevates ITGB4 expression and activates PI3K/AKT signaling, emphasizing the tumor‐suppressive function of m^6^A in this context [167]. ALKBH5 is downregulated in gastric cancer, where it suppresses metastasis through m^6^A‐dependent regulation of targets such as PKMYT1 and WRAP53. Its loss enhances stability and translation of these transcripts, activating downstream oncogenic pathways [168, 169]. Similarly, in papillary thyroid cancer or prostate cancer, reduced FTO expression leads to increased m^6^A levels on *CDH12* (cadherin 12) or *DDIT4* mRNA, which is recognized by IGF2BP2/3, enhancing its stability and driving EMT‐mediated invasion [170, 171]. In breast cancer, readers such as YTHDC1 and YTHDF3 facilitate TGF‐β‐induced EMT and Notch2 translation, respectively, promoting aggressive phenotypes [172, 173]. These studies collectively underscore that the functional outcomes of m^6^A modifications are shaped by cellular context, specific enzyme expression, and interactions with different RNA‐binding proteins. The emerging pattern suggests that m^6^A writers and erasers do not operate in isolation but form intricate networks that converge on key oncogenic pathways such as Wnt/β‐catenin. Further investigation is needed to elucidate how tissue‐specific expression of m^6^A components and their isoforms fine‐tune metastatic behaviors, which may pave the way for more precise RNA epitranscriptomic‐based therapeutics.

The emergence of therapeutic resistance in various cancers is frequently linked to the dysregulation of m^6^A RNA modification. In KRAS‐mutant colorectal cancer, elevated ALKBH5 promotes Cetuximab resistance by erasing m^6^A marks on *FOXM1* transcripts, thereby enhancing its expression and subsequently activating Wnt/β‐catenin signaling [174]. A parallel mechanism of m^6^A‐erase‐driven chemoresistance is observed in lung cancer, where KRAS mutants modulate ALKBH5 to confer platinum resistance [175]. In hepatocellular carcinoma (HCC), the writer METTL3 contributes to oxaliplatin resistance through YTHDF2‐mediated decay of *TRIM21* (tripartite motif‐containing 21) mRNA, attenuating its tumor‐suppressive effects [176]. Similarly, readers of m^6^A modifications also play divergent roles in drug resistance. YTHDF1 facilitates cisplatin resistance in ovarian cancer by stabilizing *FZD7* (frizzled class receptor 7) mRNA and amplifying Wnt/β‐catenin signaling, whereas in HCC, the YTHDF2‐c‐Jun axis, hijacked by CRTC2, drives lenvatinib resistance via enhanced translational efficiency [177, 178]. Another reader, IGF2BP3, promotes cisplatin resistance in bladder cancer by stabilizing *CDK6* (cyclin‐dependent kinase 6) mRNA in an m^6^A‐dependent manner, highlighting how different readers can converge on cell cycle regulation to sustain chemoresistance [179]. Collectively, these studies reveal that m^6^A‐mediated resistance often operates through the regulation of key signaling pathways (e.g., Wnt/β‐catenin) and cell cycle mediators, yet the specific writers, erasers, readers, and downstream targets that are involved are highly specific to both the cancer type and drugs. This mechanistic diversity suggests that targeting m^6^A machinery for reversing therapy resistance will require highly tailored approaches, including a careful consideration of the genomic background (e.g., KRAS mutation status) and the specific epitranscriptomic landscape of each tumor type.

#### 5.2.2 m^6^A in Tumor Microenvironment and Immune Evasion

The immune system serves as a primary defense mechanism against cancer through a process termed immunosurveillance, which identifies and eliminates dysregulated cells to suppress tumor formation and dissemination. Despite this protective monitoring, malignant cells develop sophisticated capabilities to evade immune detection, a critical factor enabling cancer progression. Their evasion strategies are multifaceted, involving downregulation of major histocompatibility complex (MHC) molecules, secretion of immunosuppressive cytokines such as TGF‐β and IL‐10, recruitment of regulatory T cells (Tregs) and myeloid‐derived suppressor cells (MDSCs), and direct inhibition of effector T cell activity. Within the tumor microenvironment (TME), these processes act synergistically to dampen antitumor immunity, fostering conditions conducive to tumor survival, proliferation, and metastasis. A particularly significant mechanism of immune suppression arises from the activation of checkpoint pathways, including PD‐1/PD‐L1 (programmed cell death‐ligand 1), which inhibit T cell function and accelerate disease progression. Thus, the ability to evade immune destruction is not merely a peripheral feature but a central driver of oncogenesis. Understanding these pathways has provided a compelling rationale for immunotherapy approaches that aim to disrupt immune evasion and restore antitumor responses.

The dynamic changes of RNA m^6^A during cancer formation and progression contribute to quick adaptation to microenvironmental changes. In hepatocellular carcinoma (HCC), YTHDF2 is upregulated and drives immune suppression and angiogenesis by enhancing the translation of ETV5 (ETS variant transcription factor 5), which transactivates PD‐L1 and VEGFA [180]. Similarly, in gastric cancer (GC), IGF2BP3 stabilizes *LDHA* (lactate dehydrogenase A) mRNA to augment lactate production, thereby inhibiting CD8^+^ T‐cell function [181], while METTL5 (a novel m^6^A methyltransferase)‐mediated m^6^A modification stabilizes *NRF2* mRNA to suppress ferroptosis and attenuate T‐cell cytotoxicity, highlighting how both readers and writers can modulate metabolic pathways to foster an immunosuppressive niche [182]. Conversely, the demethylase ALKBH5 exhibits context‐dependent roles: in glioblastoma (GBM), hypoxia‐induced ALKBH5 stabilizes lncRNA NEAT1 to enhance CXCL8 secretion and macrophage recruitment, whereas in intrahepatic cholangiocarcinoma (ICC), it posttranscriptionally sustains PD‐L1 expression by erasing m^6^A marks, impairing T‐cell responsiveness [183, 184]. Beyond immune checkpoint regulation, m^6^A modifiers also influence matrix remodeling and stromal signaling; for instance, in pancreatic ductal adenocarcinoma (PDAC), stiffness‐stabilized IGF2BP2 upregulates sphingomyelin synthesis to promote PD‐L1 membrane localization, and in breast cancer (BC), the VIRMA‐HNRNPC axis increases DDR1 transcription to align collagen fibers and exclude antitumor immune cells [185, 186]. In summary, further investigation is needed to determine how best to integrate epitranscriptomic inhibitors with existing immunotherapies to overcome resistance. Importantly, the tissue‐specificity of these mechanisms suggests that therapeutic targeting (e.g., inhibiting ALKBH5 in ICC or YTHDF2 in HCC) may require precision strategies tailored to the cancer and TME context.

### 5.3 m^6^A‐Regulated in Neurological and Psychiatric Disorders

The nervous system has more m^6^A than other organs, indicating its crucial role in brain function [187]. Several studies reported that FTO reduced neuronal damage in ischemic stroke. One study showed that FTO enhanced the expression of SIRT6 protein in an m^6^A‐YTHDF2‐dependent manner, which subsequently activated AMPK/PGC1α/AKT signal transduction and induced mitochondrial autophagy [188]. In two independent studies, FTO has been found to directly inhibit the expression of DRP1 protein by reducing deposition of m^6^A modification in its transcripts, or indirectly inactivate the DRP1 signal by inhibiting FYN expression via the m^6^A modification, thus reducing mitochondrial dysfunction in cerebral ischemia‐reperfusion injury [189, 190]. Furthermore, silencing FTO increased the level of methylated *Drp1* mRNA and inhibited its degradation, leading to mitochondrial fragmentation. While overexpression of FTO reversed these effects and improved hepatic ischemia‐reperfusion injury [191]. Additionally, total m^6^A level was significantly decreased, and FTO expression was increased in Parkinson's disease (PD) models in vivo and in vitro [192]. FTO promoted the stabilization of ataxia telangiectasia mutated (ATM) mRNA in dopaminergic neurons. Knockdown of FTO concomitantly suppressed upregulation of α‐Synuclein (α‐Syn) and downregulation of tyrosine hydroxylase, and alleviated neuronal death in PD models.

In parallel, the m^6^A writers and erasers cooperatively regulate cerebral ischemia‐reperfusion injury. METTL3 was responsible for the m^6^A RNA modification of maternally expressed 3 (MEG3), which subsequently regulated SIRT2 expression in an HNRNPA1‐dependent manner, resulting in oxidative stress in ischemic stroke [193]. METTL3 is also a risk factor for the occurrence of Alzheimer's disease (AD). The expression of METTL3 was increased in specific brain regions of 5xFAD mice and postmortem AD patients. METTL3 could enhance the decay of *Lingo2* mRNA through m^6^A modification, and the administration of METTL3 inhibitor STM2457 significantly alleviated the neuropathology and behavioral deficits of AD mice [194]. Similarly, single‐cell and spatial transcriptome have revealed that protein tyrosine phosphatase receptor type G prevented mitophagy‐mediated neuronal death in AD by activating another methyltransferase VIRMA [195].

In other neurological diseases, downregulation of METTL3 prevented the translocation of TNF receptor‐associated factor 6 (TRAF6) to mitochondria in microglia and subsequent activation of the TRAF6/ECSIT pathway, leading to reduced mitochondrial ROS production and protecting the balance of sympathetic activity against postmyocardial infarction [196]. Experimental depletion of METTL14 has allowed researchers to dissect the contribution of m^6^A‐mitochondria regulation in the pathogenesis of neurological disorders. In accordance with the model described above, the deficiency of METTL14 impaired mitochondrial function by translational efficiency of mitochondrial complex subunit RNAs in an m^6^A‐dependent manner in neuronal cells [197]. As outlined above, the roles of m^6^A regulation in neural development and neurological disorders are very important, and the consequences of m^6^A dysregulation are therefore severe.

m^6^A RNA methylation plays a significant role in the regulation of mood and stress, with growing evidence linking it to psychiatric phenotypes. FTO was downregulated in the hippocampus of patients with major depressive disorder (MDD) and mouse models of depression [198]. Suppressing *Fto* expression in the mouse hippocampus results in depression‐like behaviors in adult mice, whereas overexpression of FTO leads to rescue of the depression‐like phenotype. Adrenoceptor beta 2 (Adrb2) mRNA, as a target of FTO, can rescue the depression‐like behaviors in mice and spine loss induced by hippocampal *Fto* deficiency.

### 5.4 m^6^A‐Regulated in Inflammatory and Autoimmune Diseases

Emerging evidence shows that m^6^A modification is linked to inflammation‐related diseases [199]. The literature is becoming inundated with evidence that METTL3 is important for m^6^A regulation in inflammation. During oxidized low‐density lipoprotein‐induced monocyte inflammation, METTL3 and YTHDF2 synergistically modified the PGC1α transcripts, mediated its degradation, decreased PGC1α protein levels, and reduced cellular ATP production and oxygen consumption rate. This subsequently increased the accumulation of cellular reactive oxygen species, as well as the levels of pro‐inflammatory cytokines in inflammatory monocytes [200]. The expression level of methyltransferase METTL3 is significantly increased in M1 macrophages, which enhances the mRNA stability and protein expression of hepatoma‐derived growth factor (HDGF) through m^6^A RNA methylation, thereby contributing to M1 macrophage polarization [201]. These studies suggested that METTL3‐ m^6^A directly participates in the regulation of inflammatory cells.

In murine sepsis models, STM2457 (a highly selective METTL3 inhibitor) administration or *Mettl3* conditional knockout in type II alveolar epithelial cells significantly attenuated sepsis‐induced acute lung inflammation. This protective effect was attributed to decreased m^6^A modification levels in acyl‐CoA synthetase long chain family member 4 (ACSL4) and *HIF1α* transcripts. Mechanistically, METTL3 prolonged m^6^A‐modified *ACSL4* or *HIF1α* mRNA lifespan in an m^6^A‐YTHDC1/IGF2BP2‐dependent manner, leading to mitochondria‐associated ferroptosis [202, 203]. Parallel findings in lipopolysaccharide (LPS)/cisplatin‐treated HK‐2 cells revealed that METTL3 or WTAP knockdown alleviated ferroptosis [204, 205, 206]. Mechanistically, WTAP downregulation directly enhanced lamin B1 (LMNB1) expression via m^6^A‐mediated mechanisms. METTL3 deficiency, on the one hand, reduced m^6^A methylation of *MDM2*, thereby reducing YTHDF1‐mediated *MDM2* mRNA translation, indirectly leading to inhibiting LMNB1 expression. On the other hand, METTL3 deficiency destabilized SREBP1c transcripts in an m^6^A‐IGF2BP3‐dependent manner, thus indirectly restoring OPA1‐mediated mitochondrial dysfunction. Notably, YTHDF1 ablation exacerbated function in the process of fulminant hepatitis by diminishing m^6^A‐dependent translation of milk fat globule EGF factor 8 (MFG‐E8) [207]. Besides, human cytomegalovirus infection induced vascular endothelial inflammatory injury, which might be largely related to the abnormal increase of m^6^A modification caused by METTL3. On the one hand, the METTL3‐specific inhibitor restored the expression of ubiquitin carboxyl terminal hydrolase‐L1 via an m^6^A‐HNRNPD‐dependent mechanism, thus alleviating the inflammatory injury of vascular endothelial cells [208]. On the other hand, METTL3‐m^6^A‐YTHDF3 increased the translation and expression of mitochondrial calcium uniporter, and these increments could be counteracted by ALKBH5 overexpression [209]. As mentioned above, METTL3 is a potential therapeutic target in inflammatory diseases. Generally, METTL3 positively correlated with inflammatory diseases [210], and it is meaningful to explore the specific inhibitors of METTL3 for inflammatory diseases. While METTL3 negatively correlated with some inflammatory diseases, such as periodontitis, this correlation cannot be ignored.

The interplay between inflammation and autoimmunity is a cornerstone of much chronic pathology, where persistent inflammatory signaling bypasses homeostatic checkpoints to trigger self‐reactive immune responses. As a critical posttranscriptional regulator, m^6^A methylation orchestrates the delicate balance between pro‐ and anti‐inflammatory pathways. Autoimmune diseases occur when there is an imbalance in m^6^A modification in immune cells, triggering immune system malfunction.

Recent evidence has consistently shown that m^6^A modifiers, including METTL3, FTO, YTHDF1, and YTHDF2, are key drivers of systemic lupus erythematosus (SLE). A significant commonality across recent research is the focus on B‐cell lineage malfunctions, where m^6^A modifiers dictate the expansion and differentiation of effector subsets. For instance, the demethylase FTO is significantly upregulated via toll‐like receptor 7‐myeloid differentiation primary response protein 88 (TLR7‐MyD88) signaling, where it targets ATPase H^+^ transporting V1 subunit G1 (ATP6V1G1) to enhance vacuolar H^+^‐ATPase (V‐ATPase) activity and lysosomal autophagy, thereby metabolically shaping the expansion of extrafollicular age‐associated B cells (ABCs) [211]. Similarly, the differentiation of plasma cells (PCs) is critically dependent on the m^6^A‐mediated stabilization of the transcription factor IRF4; this is achieved either through the reader YTHDF1 increasing mRNA stability or the writer METTL3 promoting its expression, both of which lead to heightened autoantibody generation and PCs infiltration‐mediated kidney damage of SLE [117, 212]. While B cell‐centric studies emphasize metabolic and transcriptional stability, a complementary perspective highlights the role of m^6^A in the T cell compartment, specifically T_H_17 cell‐driven autoimmunity. Specifically, the lncRNA ALKBH3‐AS1 acts as a molecular scaffold to recruit the reader YTHDF2, facilitating the decay of *SMAD3* mRNA to inhibit Th17 differentiation (a pathway that is characteristically suppressed in SLE patients) [213].

m^6^A modification exhibits a central regulatory role in the multidimensional pathogenesis of rheumatoid arthritis (RA). The abnormal expression of m^6^A modifiers such as METTL3, METTL14, ALKBH5, IGF2BP2, and IGF2BP3 is closely related to RA disease activity, synovial hyperplasia, and inflammatory cytokine secretion. In addition, dysregulated m^6^A levels are correlated with disease activity, and therapeutic targeting of these pathways in vivo yields benefits in arthritis models, underscoring their clinical potential. However, targeting different cell types and molecular targets, these studies reveal complementary or distinct modes of regulation. In terms of synovial lesions, the upregulation of METTL3 and IGF2BP3 significantly drives the “tumor‐like” characteristics of synovial fibroblasts: METTL3 enhances the stability of *SLC7A11* (solute carrier family 7 member 11) mRNA through IGF2BP2‐mediated m^6^A modification, thereby promoting cell proliferation and invasion by inhibiting ferroptosis [214]; while IGF2BP3 triggers pathological proliferation and inflammatory responses through RASGRF1 (Ras protein specific guanine nucleotide releasing factor 1)‐mediated mTORC1 activation [215]. At the level of immune regulation, research presents a more complex landscape. On the one hand, PIWI‐interacting RNAs (such as piENOX2) can serve as an upregulating factor to downregulate ALKBH5, thereby regulating the m^6^A level of *Itga4* and inducing macrophages to polarize toward the pro‐inflammatory M1 type through the PI3K‐AKT signaling pathway [216]. On the other hand, unlike the pathogenic role of METTL3, METTL14 shows reduced expression in peripheral blood mononuclear cells (PBMCs) of RA patients, and its level is negatively correlated with the disease activity score (DAS28) [217]. The loss of METTL14 reduces the mRNA stability and translation efficiency of the NF‐κB inhibitor TNFAIP3, thereby releasing the inhibition of inflammatory factors such as IL‐6 and IL‐17, and aggravating systemic inflammation.

### 5.5 m^6^A‐Regulated in Organ Injury and Repair

Research in recent years has not only revealed the basic role of m^6^A in maintaining the homeostasis of healthy organs, but also profoundly clarified how its dysregulation leads to fibrosis progression and acute injury. Chronic organ injury leads to cellular and molecular responses that cause fibrosis [218]. Fibrosis increases the incidence and mortality of various organ diseases, including the liver, heart, kidney, and lung [219]. As part of a degenerative process stemming from dysregulated tissue repair, this pathological state involves the excessive deposition of extracellular matrix by activated myofibroblasts in response to sustained injury [220]. Conversely, acute organ injury typically results from rapid‐onset damage such as ischemia‐reperfusion, toxin exposure, or trauma. The ensuing cellular stress and necrosis may either be reversed by regenerative mechanisms or, if the damage is overwhelming, tip the balance toward a pathological fibrotic cascade.

#### 5.5.1 m^6^A in Fibrosis

In hepatic fibrosis, the proliferation and migration of hepatic stellate cells (HSC) resulted from the decrease of ALKBH5 expression. Mechanically, ALKBH5 mediated m^6^A demethylation in *Drp1* mRNA 3’UTR, while the deletion of ALKBH5 hastened the decay of *Drp1* mRNA in an m^6^A‐YTHDF1‐dependent manner, and promoted mitochondrial fission [221]. Another independent study found that FTO and ZC3H13 coordinately regulated m^6^A levels of nuclear receptor subfamily 1 group D member 1 (NR1D1) mRNA, and the m^6^A‐tagged *NR1D1* mRNA was degraded by YTHDC1, which further inhibited the phosphorylation of DRP1^S616^, resulting in decreased mitochondrial fission and stimulated HSC activation [222]. Additionally, m^6^A modification on peroxiredoxin 3 (PRDX3) mRNA facilitates its interaction with YTHDF proteins; YTHDF3, in particular, promotes PRDX3 translation, inhibiting HSC activation via the TGF‐β1/Smad2/3 pathway [223]. Given that m^6^A is regulated by multiple regulators and their substrate preference, it is straightforward to understand why the differential abundance of m^6^A in different transcripts occurs during HSC activation.

Cardiac fibrosis is commonly regarded as the ultimate pathway for various heart diseases and is also modulated by m^6^A‐dependent mechanisms. Growth arrest‐specific 5 (GAS5)/androgen receptor (AR) directly interacted with Drp1/Decr1, respectively, and inhibited Drp1/Decr1‐mediated mitochondrial fission/mitochondrial lipid oxidation [224, 225]. Mechanistically, m^6^A methyltransferases METTL3 and WTAP mediated m^6^A methylation on lncRNA *GAS5* and *AR* mRNA, respectively, and both depended on YTHDF2 to induce their degradation. Furthermore, using hypoxia‐ischemia and TGF‐β1‐induced fibrosis cell models, Huang et al. found that inhibition of METTL3/14 could effectively reverse cardiomyocyte death by inhibiting myofibrillar conversion, which was linked to a reduction in *Drp1* mRNA translation efficiency via a METTL3‐m^6^A‐dependent mechanism [226].

m^6^A regulation in lung and kidney fibrosis has also attracted the attention of researchers. ALKBH5 is highly expressed in lung tissue [227], and its m^6^A demethylase activity is inhibited by SUMOylation [228]. In the models of pulmonary fibrosis, 1‐nitropyrene promoted ALKBH5 SUMO and upregulated m^6^A modification of F‐box and WD repeat domain containing 7 (FBXW7) mRNA in a YTHDF1‐dependent manner [229]. In the pathogenesis of renal fibrosis, METTL3 mediated m^6^A hypermethylation of *β‐catenin* mRNA, and boosted β‐catenin expression, thus promoting renal fibrosis progression by promoting its downstream profibrotic genes expression [230]. STM2457‐induced METTL3 inhibition attenuated the extent of renal fibrosis in vivo by reducing TGFβ‐induced fibrosis marker expression in HK2 cells [231]. As a result, some m^6^A regulators exhibit a dual role in promoting and inhibiting fibrosis [232], suggesting sophisticated spatiotemporal‐specific functional compartmentalization within the epitranscriptomic network in renal fibrosis. Moreover, m^6^A modification is involved in other pathways, such as TGF‐β signaling and inflammation. Thus, more investigation is required before fibrosis therapy can use m^6^A‐related medicines.

#### 5.5.2 m^6^A in Acute Injury

METTL3‐IGF2BP2 mediates m^6^A modification on DLG‐associated protein 5 (Dlgap5) mRNA, increasing its stability, and then DLGAP5 promotes pyroptosis through NF‐κB‐dependent NOD‐like receptor family, pyrin domain‐containing protein 3 (NLRP3) inflammasome activation and directly enhances inflammasome structure formation and assembly, thus exacerbating acute liver injury [233]. During the process of concanavalin A (ConA)‐induced liver injury, hepatic YTHDF1 protein decreased rapidly, and YTHDF1‐deficient mice were more susceptible to ConA‐induced liver injury, accompanied by an intensification of the inflammatory storm and exacerbating the liver inflammatory response through the ERK and NF‐κB pathways [234].

Gao et al. showed that both *Alkbh5* whole‐body knockout and myocardial‐specific knockout mice were more susceptible to doxorubicin‐induced cardiotoxic injury; meanwhile, ALKBH5 deficiency led to mitochondrial damage, including the destruction of mitochondrial architecture, accumulation of ROS, and a decrease of mitochondrial membrane potential, whereas ALKBH5 overexpression rescued mitochondrial dysfunction and attenuated cardiotoxic injury [235]. In addition, they found that ALKBH5 downregulated the expression of RAS protein activator like 3 (RASAL3) in an m^6^A‐dependent manner through reduced *Rasal3* mRNA lifespan, protecting against doxorubicin‐induced myocardial mitochondria dysfunction and cardiotoxic injury. These novel findings contribute to the search for potential intervention targets to lessen the cardiac damage of anthracycline drugs in cancer patients. Consistently, FTO has a protective effect on mitochondria in cardiomyocytes [236]. FTO depletion elevated m^6^A modification levels in BCL2‐interacting protein 3 (BNIP3) transcripts and destabilized its mRNA in an m^6^A‐YTHDF2‐dependent manner, thus suppressing mitophagy and exacerbating sepsis‐induced cardiac injury [237, 238]. As a downstream factor of circ‐ZNF609, the expression of FTO was upregulated after circ‐ZNF609 was inhibited, thus blocking METTL14‐mediated the increase of RNA m^6^A methylation level in the heart of doxorubicin‐treated mice and improving mitochondrial non‐heme iron overload [239]. During the fetal stage, myocardial cells promote the formation of the heart through cardiomyocyte proliferation. However, in the later stages of cardiac development, the development of the heart occurs through hypertrophic growth of individual muscle cells, rather than additional cell division, because myocardial cells lose the ability to divide [240]. METTL3 is important for cardiomyocyte proliferation and remodeling. Although cardiomyocyte‐specific METTL3 knockout mice did not present the impairment of cardiac morphology or function at 3 months old, there appeared to be a reduction in cardiomyocyte cross‐sectional area and a decrease in overall cardiac function consistent with a progression toward heart failure at 8 months old [241]. A recent study showed that METTL3 deficiency distinctly reduced the abundance of mitochondrial fatty acid oxidation genes (*Acadm*, *Mlycd*, and *Nudt7*) and mitochondrial electron transport chain genes (*Atp5o* and *Coq9*), which resulted in decreased oxygen consumption rates in 3‐month‐old cardiomyocyte‐specific METTL3 knockout mice [242]. These findings suggested that METTL3 deficiency mediates cardiac mitochondrial dysfunction via an m^6^A‐dependent manner during aging, but its precise mechanisms require further investigation. Of interest, METTL3 exhibited a specific role in myocardial regulation when faced with different situations. Jiang et al. [243] reported that injection of the AAV9‐shMETTL3 virus improved cardiac remodeling and dysfunction after myocardial infarction in mice. One possible explanation for this result is that METTL3 rapidly triggered the maturation of pri‐miR‐503 and promoted exosomal miR‐503 biogenesis via an m^6^A‐HNRNPA2B1‐dependent mechanism in cardiac endothelial cells. And exosomal miR‐503 mediated cardiomyocyte damage by triggering mitochondrial metabolic perturbance. Endothelial METTL3 deficiency ameliorated cardiac ischemic injury, but it was aborted by miR‐503 inhibition [244]. In addition, another writer, WTAP, has also been reported to be involved in myocardial ischemia‐reperfusion injury, which could downregulate the stability of lncRNA *Snhg1* in an m^6^A‐YTHDF2‐dependent manner, thus inhibiting miR‐361‐5p/OPA1‐mediated mitochondrial fusion [245].

METTL3 is upregulated in acute lung injury, exerting influence on cellular damage and inflammation by modulating signaling pathways such as ACSL4 [202]. METTL3 promotes m^6^A methylation of spleen‐associated tyrosine kinase (SYK) mRNA, enhancing its stability and transcription, along with SYK phosphorylation and downstream ERK/MEK activation, thereby inducing acute lung injury [246]. Similarly, METTL3/METTL14‐mediated m^6^A modification is enriched in *ACSL4* or *NRF2*, and their mRNA stability is regulated through an YTHDC1/YTHDF2‐dependent pathway [202, 247]. Inhibition of METTL3 or METTL14 through knockdown effectively suppresses septic hyperlactate‐induced ferroptosis in alveolar epithelial cells and mitigates lung injury in septic mice. METTL14‐catalyzed *NLRP3* mRNA m^6^A methylation enhances the stability of *NLRP3* mRNA in an IGF2BP2‐dependent manner in acute lung injury [248]. Obesity‐induced upregulation of FTO inhibits the expression of miR‐192, thereby promoting macrophage activation and aggravating LPS‐induced acute lung injury [249].

Acute exposure to high concentrations of lead leads to renal damage and significant upregulation of m^6^A methylation, which is mainly mediated by METTL3 [250]. Hexokinase domain‐containing 1 (HKDC1) is a direct target of METTL3, and m^6^A modification mediates the upregulation of *HKDC1* mRNA and protein levels, thereby promoting renal injury and inflammation. METTL3 was upregulated in ischemic acute kidney injury (AKI) models [251]. Mechanistically, METTL3 mediates m^6^A modification of *TIFA* (TRAF interacting protein with forkhead associated domain) mRNA, which is recognized by IGF2BP2 to enhance mRNA stability. Similarly, the crosstalk between METTL3‐mediated m^6^A modification and ferroptosis in AKI has also been elucidated. Inhibition of METTL3 expression in vivo and in vitro alleviated the damage and ferroptosis in renal tubular cells [252]. Mechanistically, heme oxygenase 1 (Hmox1/HO‐1) was the METTL3 target, and IGF2BP3 could be used as a reader to bind to the methylated site of *Hmox1* mRNA to maintain its stability. Additionally, METTL3‐mediated SREBP1c upregulation contributes to AKI through disrupting mitochondrial energy metabolism via transcriptionally suppressing YME1L1 [204]. Decreased circAASS expression and its association with impaired mitochondrial function in TECs, followed by more severe renal fibrosis, are observed in AKI patients. Mechanistically, IGF2BP2 suppresses circAASS biogenesis by binding to intronic sequences in the AASS pre‐mRNA [253]. Renal tubular‐specific YTHDF1 knockout mice exhibit heightened AKI severity when contrasted with their wild‐type counterparts [254].

## 6 m^6^A‐Regulated Mitochondrial Remodeling: A Dedicated Deep Dive

**FIGURE 5:**
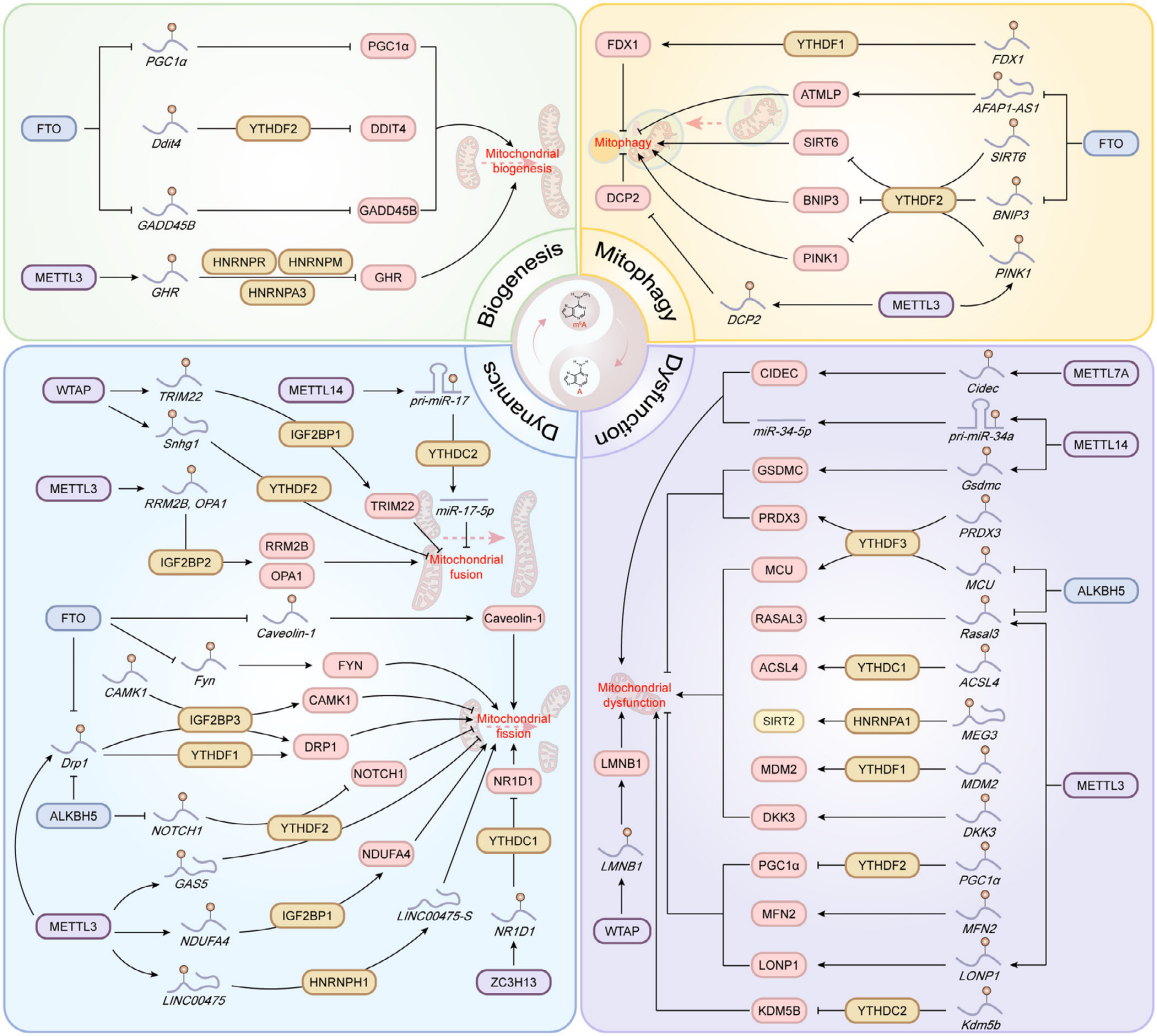
Regulatory mechanism of N^6^‐methyladenosine (m^6^A)‐related enzymes in mitochondrial remodeling. m^6^A modifiers regulate key factors involved in the regulation of mitochondrial activity, including mitochondrial biogenesis, dynamics (fission and fusion), dysfunction, and mitophagy. In this context, dysregulated m^6^A deposition alters the stability, translation, or processing of transcripts and their associated effectors, thereby reshaping mitochondrial homeostasis. Furthermore, these m^6^A‐dependent transcripts influence signaling pathways linked to mitochondrial dysfunction, ultimately affecting cellular homeostasis and disease‐related phenotypes.

To date, m^6^A modification has been shown to affect various processes by regulating mitochondrial homeostasis, while the dysregulation of m^6^A modification is associated with mitochondrial disorders in various diseases (Figure 5).

### 6.1 m^6^A in Mitochondrial Biogenesis

Mitochondrial biogenesis is a highly “bicentric controlled” (synchronously regulated by mtDNA and nuclear DNA) process to produce new mitochondria from existing mitochondria. PGC1α has been regarded as a central regulator of mitochondrial biogenesis due to its capacity to increase the expression and activity of multiple key transcription factors [255]. Briefly, PGC1α activates transcription of nucleus‐encoded mitochondrial genes. Then, nucleus‐encoded mitochondrial proteins translocate to targeted mitochondria and complete assembly. Meanwhile, transcription of the TFAM, a major mitochondrial transcription factor, activates mtDNA transcription and increases mtDNA copy number. Finally, mitochondrial phospholipids are synthesized, promoting mitochondrial biogenesis [256]. The impairment of mitochondrial biogenesis has been implicated in a variety of human disorders such as aging, metabolic diseases, neurodegeneration, and cancer [257].

In renal cell carcinoma, genes related to mitochondrial biogenesis (*Pgc1α* and *Tfam*) and oxidative phosphorylation (*Cox5a*, *Atp5g1*, *Atp5a1*, and *Cycs*) were specifically upregulated in FTO overexpression cells, while the expression of genes regulating mitochondrial fission and fusion was not changed in FTO overexpression cells. FTO restored mitochondrial activity and increased mitochondrial biogenesis by stabilizing *PGC1α* mRNA in an m^6^A‐dependent manner, which exerted an anticarcinogenic effect [258]. Further investigation into myogenesis has identified growth arrest and DNA damage‐inducible 45B (*GADD45B*) as a downstream target of FTO. Mechanistically, FTO deficiency leads to hypermethylation and the subsequent m^6^A‐dependent degradation of *GADD45B* mRNA. This loss of *GADD45B* attenuates the p38 MAPK‐PGC1α signaling axis, thereby suppressing mitochondrial biogenesis [259]. Similarly, FTO has been shown to reduce m^6^A levels of DNA damage‐induced transcript 4 (Ddit4) mRNA, enhancing its stability via YTHDF2‐mediated recognition; this upregulates DDIT4 protein expression and bolsters PGC1α‐mediated mitochondrial biogenesis [260]. Conversely, METTL3‐dependent m^6^A modification downregulates GHR mRNA expression to impair mitochondrial function by inhibiting mitochondrial biogenesis [261]. Beyond direct transcript stability, mitochondrial homeostasis is regulated by complex interactions between circular RNAs and m^6^A readers. For instance, the expression of *circAASS* is negatively correlated with the reader IGF2BP2. Nuclear‐localized *circAASS* directly interacts with the PGC1α protein, sequestering it to prevent ubiquitin‐mediated degradation [253]. This protein‐level stabilization bypasses typical RNA‐level decay pathways to promote mitochondrial biogenesis, thereby alleviating renal injury and fibrosis in tubular epithelial cells. Interestingly, the regulation of circAASS by IGF2BP2 occurs independently of m^6^A.

### 6.2 m^6^A in Mitochondrial Dynamics

The mitochondrial network maintains dynamic balance through DRP1‐mediated fission (responsible for mitochondrial generation and damaged degradation) and MFN1/2 and OPA1‐driven fusion (aimed at enhancing activity and repairing damage). This precise regulation of fission and fusion is crucial to cell function. Defects in its key proteins can lead to abnormal mitochondrial structure and induce a variety of serious diseases, such as cardiovascular, metabolic, and neurodegenerative diseases.

NDUFA4 encodes a subunit of the electron transport chain complex belonging to the mitochondrial respiratory chain and is used to generate ATP [262]. Silencing METTL3 could decrease the m^6^A level of NADH dehydrogenase (ubiquinone) 1α subcomplex 4 (NDUFA4) mRNA 3′UTR region, which attenuated the stabilizing effect of IGF2BP1 on *NDUFA4* mRNA and reduced the abundance of NDUFA protein, thereby inhibiting mitochondrial fission [263]. METTL3 induced LINC00475‐S production by m^6^A‐modified spliced LINC00475 and then promoted mitochondrial fission in glioma cells by inhibiting the expression of macrophage migration inhibitory factor. Pull‐down combination LC/MS and RIP detection showed that m^6^A‐recognized protein HNRNPH1 bound to LINC00475 in GYR and GY domains and promoted LINC00475 splicing [264]. FTO depletion significantly decreased the protein expression of Pink1, phosphorylated Parkin1, and phosphorylated MFN2, resulting in increased mitochondrial fission in AGS and SGC‐7901 cells. In contrast, caveolin‐1 depletion could remarkably reverse these effects. Further research found that FTO directly targeted *caveolin‐1* mRNA and promoted its degradation [265]. Similarly, FTO overexpression inhibited FYN expression via the m^6^A modification to inactive Drp1 signaling, thus reducing mitochondrial fission [190]. Decreased ALKBH5 expression was accompanied by high mitochondrial fission. The downregulation of ALKBH5 elevated m^6^A levels in the 3’UTR of notch receptor 1 (Notch1) mRNA and facilitated *Notch1* mRNA degradation through a mechanism involving m^6^A and YTHDF2, thereby promoting *Drp1* transcription and mitochondrial fission [266]. Yuan et al. showed that high glucose treatment also reduced the levels of IGF2BP3 in HK‐2 cells. And they found that IGF2BP3 extended the lifetime of calcium/calmodulin‐dependent protein kinase 1 (CAMK1) mRNA by the recognition of the m^6^A region, which could increase the abundance of CAMK1 protein and suppress mitochondrial fission to protect against high glucose‐induced kidney injury [267].

The increased of WTAP promoted the protein expression of tripartite motif containing 22 (TRIM22) in a m^6^A‐IGF2BP1‐dependent manner, then TRIM22 interacted with OPA1 and disrupted mitochondrial fusion [268]. Low expression of METTL14 triggered a decrease in m^6^A modification, thereby inhibiting the decline of pri‐miR‐17 and increasing the expression of miR‐17‐5p by reducing YTHDC2's recognition of the “GGACC” binding site; miR‐17‐5p directly bound to the 3’untranslated region (3’UTR) of Mfn2, resulting in decreased mitochondrial fusion [269]. The METTL3‐IGF2BP2 axis upregulated the expression of ribonucleoside‐diphosphate reductase subunit M2B (RRM2B), a p53‐induced ribonucleotide reductase subunit with antioxidant potential and OPA1 in an m^6^A‐dependent manner, thereby triggering glutathione production and promoting mitochondrial fusion [270].

### 6.3 m^6^A in Mitophagy

Mitophagy is a process in which cells selectively clear damaged or redundant mitochondria to maintain homeostasis through two mechanisms: ubiquitin‐mediated (PINK1/Parkin pathway) or receptor‐mediated (such as BNIP3, FUNDC1, etc.). This precise quality control is essential for maintaining cellular health, and once this function is disrupted, the accumulation of damaged mitochondria becomes the causative core of a variety of chronic diseases, including metabolic disorders, neurodegenerative diseases, and cancer.

METTL3 is a marker of poor prognosis of SCLC and is highly expressed in chemotherapy‐resistant SCLC cells. Mechanistically, METTL3 induced m^6^A methylation of decapping protein 2 (DCP2) mRNA and caused its degradation, thereby promoting mitophagy through the Pink1‐Parkin pathway, leading to chemotherapy resistance, while STM2457 (METTL3 inhibitor) could reverse SCLC chemotherapy resistance [271]. METTL3 and YTHDF2 synergistically accelerated *PINK1* mRNA decay in an m^6^A‐dependent manner, resulting in impairment of mitophagy and renal cell injury [272]. Another m^6^A recognition protein, YTHDF1, was found to be upregulated in glioma tissues [273]. It was found that c‐MYC might be the upstream regulatory factor of YTHDF1, while ferredoxin 1 (FDX1) might be its downstream target; c‐MYC could upregulate FDX1 and inhibit mitophagy in glioma cells through YTHDF1 [274]. It is worth noting that lncRNA actin filament‐associated protein 1 antisense RNA 1 (AFAP1‐AS1) encodes a conservative peptide of 90 amino acids located in mitochondria, named lncRNA AFAP1‐AS1 translated mitochondrial localization peptide (ATMLP), which is not lncRNA, but a polypeptide that promotes the malignant progression of non–small cell lung cancer (NSCLC). Mechanistically, the translation of ATMLP is controlled by adenine m^6^A methylation at the AFAP1‐AS11313 site. FTO overexpression or AFAP1‐AS11313 m^6^A mutation significantly reduced AFAP1‐AS1 translation. ATMLP is bound to the 4‐nitrophenylphosphatase domain and nonneuronal SNAP25‐like protein homology 1 (NIPSNAP1) and inhibits its translocation from the inner to the outer membrane of mitochondria, thereby antagonizing the regulation of NIPSNAP1‐mediated cellular autolysosome formation to suppress mitophagy in NSCLC cells [275].

### 6.4 m^6^A in Mitochondrial Dysfunction

Mitochondrial dysfunction is not merely a consequence but a driver of disease progression. In the pathogenesis of renal fibrosis, METTL3 mediated m^6^A hypermethylation of dickkopf 3 (DKK3) mRNA, and boosted DKK3 expression, thus disturbing mitochondrial homeostasis by promoting MFF transcription [276]. Further studies showed that the expression of METTL3 in hippocampal tissue of the AD mouse model was downregulated, and METTL3 could enhance the expression of MFN2 through m^6^A modification, thus improving mitochondrial dysfunction [277]. Similarly, impaired METTL3‐m^6^A signal transduction at least partially led to the reduction of lon peptidase 1 (LONP1, a protease essential for protein homeostasis in the mitochondrial matrix), resulting in protein homeostasis defects and mitochondrial dysfunction in AD experimental models both in vitro and in vivo [278]. Gasdermin C (GsdmC) is highly associated with mitochondria and can maintain mitochondrial membrane potential and protect mitochondrial integrity. However, loss of METTL14 drastically reduced the methylation on *GsdmC* transcripts and abolished mitochondrial GSDMC protein synthesis in intestinal epithelial cells and colon cancer cells. The decrease in GSDMC expression disrupted mitochondrial membrane potential and triggered cytochrome c release, thereby resulting in mitochondrial dysfunction. Meanwhile, the depletion of METTL14 also mediated abnormal mitochondrial dynamics by stimulating mitochondrial FIS1 recruitment and DRP1 activation [279]. In addition, the upregulation of methyltransferase METTL14 increased the m^6^A modification of pri‐miR‐34a, which in turn promoted the expression of mature miR‐34a‐5p, leading to disequilibrium of mitochondrial dynamics by inhibiting the protein expression of SID1 transmembrane family member 2 in fatty liver. METTL14 silencing counteracted the imbalance of mitochondrial homeostasis and lipid accumulation in the livers of high‐fat diet‐fed mice [280]. With the exception of METTL3 and WTAP, silencing of METTL7A markedly alleviated high glucose‐induced mitochondrial dysfunction by inhibiting the m^6^A methylation of cell death‐inducing DNA fragmentation‐factor‐like effector C (CIDEC) mRNA in renal tubular cells [281]. YTHDC2 decreased the abundance of KDM5B protein by reducing the stability of *KDM5B* mRNA in an m^6^A‐dependent manner, which in turn ameliorated high glucose‐induced mitochondrial dysfunction by promoting the expression of SIRT3 [282].

### 6.5 Consequences for Metabolism and Bioenergetics

Current research has shown that m^6^A links cellular metabolism to mitochondrial function [283]. It directly regulates the expression of metabolic enzymes and transcriptional regulators involved in thermogenesis and glycolysis, enabling cells to adapt to nutrient availability. This regulatory layer extends to mitochondrial homeostasis, where m^6^A modifications on metabolic enzymes and nuclear‐encoded mitochondrial transcripts are involved in energy metabolism.

FTO deficiency increased energy expenditure by browning of white adipose tissue in mice, in which mitochondria play a vital regulatory role in m^6^A modification‐mediated alteration of adipose tissue thermogenesis and lipid metabolism. Loss of FTO upregulates uncoupling protein 1 (UCP1) expression and enhances mitochondrial uncoupling in adipocytes [284]. Consistent with this, the browning of white adipocytes is almost absent in mice with adipose‐specific FTO deletion. Mechanistically, FTO depletion increased the abundance of m^6^A‐modified HIF1α transcript, which can be bound by YTHDC2 for facilitating HIF1α translation, thereby positively modulating thermogenic gene expression such as *Prdm16*, *Pgc1α*, *Pparγ*, and *Ucp1*, and inducing browning and thermogenesis of white adipocytes [285]. Also, knockdown of FTO strikingly increased m^6^A levels of mitochondrial unfolded protein response markers, heat shock response 60 (Hsp60) transcript, and enhanced protein abundance of HSP60, thereby resulting in disturbance of ATF5 translocation from mitochondria to the nucleus and apoptosis in adipocytes [286]. Contrarily, Wei et al. have reported that knockdown of FTO caused an increment in m^6^A modification on perilipin5 (Plin5) mRNA, which resulted in the decreased expression of PLIN5 and a reduction in mitochondrial β‐oxidation in porcine preadipocytes [287]. This discrepancy could be attributed to a variety of factors, such as species and models. In addition, white adipose tissue beiging and mass were also regulated by METTL3 and YTHDF1. Further experiments revealed that METTL3/YTHDF1 facilitated the stability of kruppel‐like factor 9 (Klf9)/bone morphogenetic protein 8b (Bmp8b) via an m^6^A‐dependent manner to mediate adipose tissue mitochondrial uncoupling under cold stimulation [288, 289]. This ensures the coordinated expression of electron transport chain components, maintains mitochondrial membrane integrity, and regulates ROS production, thereby directly linking the epitranscriptome to cellular energy output and metabolic health.

piR‐26441 interacts with YTHDC1 and promotes the degradation of *TSFM* mRNA. Loss of TSFM reduces mitochondrial complex I activity and mitochondrial OXPHOS, leading to ovarian cancer (OC) cell mitochondrial dysfunction and increased reactive oxygen species levels, leading to OC cell DNA damage and apoptosis [290]. YTHDF1 recognizes target MeCP2 mRNA and induces its translation. Increased methylation of the SLC31A1 promoter CpG islands, recognized by MeCP2, represses its transcription, thereby exacerbating mitochondrial copper depletion and promoting glycolysis [291].

## 7 Therapeutic Targeting of the m^6^A Axis

### 7.1 Small‐molecule Inhibitors of M^6^A Modifiers

A large number of studies have shown that m^6^A plays a key role in many common disease processes, making it an attractive therapeutic target. For example, the dysregulation of METTL3 plays a driving role in diseases (e.g., fatty liver, HCC, AD, and cardiac fibrosis). Therefore, this has sparked researchers’ interest in developing small‐molecule inhibitors of m^6^A modifiers, which hold promise for providing new strategies for disease treatment (Tables 1 and 2).

**TABLE 1:** Summary of representative small‐molecule inhibitors of m^6^A writers.

		Effects					
Targets	Small‐molecule inhibitor	Enzymatic activity (IC50)	In vitro	In vivo	Potential mode of action	Selectivity	References
METTL3	STM2457	16.9 nM	Inhibited the proliferation of multiple cancer cells, including AML cell lines, neuroblastoma cells, liver cancer cell lines, CRC cells; Inhibited lung adenocarcinoma cell migration	Impeded tumor growth in liver cancer, AML, neuroblastoma, gastric cancer, and colorectal cancer xenograft mouse models; alleviated lung metastases in tail vein metastasis mouse models; ameliorated cardiac inflammation and fibrosis	Occupied SAM binding pocket	Yes	[292, 293, 294, 295, 296, 297, 298, 299]
	STC‐15	4 nM	Inhibited the proliferation of multiple cancer cells, including AML cell lines, OC cell lines, lung cancer cell lines, and FaDu cell lines	Evaluating the safety and efficacy of STC‐15 in non–small cell lung cancer, squamous cell carcinoma of the head and neck, melanoma, and endometrial cancer in patients	SAM‐competitive	/	[300, 301] NCT05584111, NCT06975293
	UZH1a	280 nM	Suppressed the viability and proliferation of Epstein‐Barr virus‐positive cancer cells	/	Occupied the pocket of the adenosine moiety of SAM	Yes	[302, 303]
	EP652	2 nM	Efficiency better than STC‐15; inhibited proliferation of multiple cancer cells, including AML cell lines, OC cell lines, lung cancer cell lines, FaDu cell lines	impeded tumor growth and prolonged survival in AML, OC, and lung cancer xenograft mouse models	Similar to STM2457 and UZH2	Yes	[292]
	EP102	/	/	Evaluating the safety and efficacy of EP102 in advanced solid tumors	/	/	NCT07163325
	Coptisine chloride	5.49 µM	Inhibited the proliferation of AML cells	Alleviated inflammatory periodontal bone loss in periodontitis mouse models	Occupied the SAM binding pocket	/	[304]
	Compound 54	54 nM	Inhibited the proliferation of AML cells	/	Occupied the SAM binding pocket	Yes	[305]
	UZH2	5 nM	Inhibited the proliferation of AML cells and PCa cells	/	SAM‐competitive	Yes	[301, 306]
	Quercetin	2.73 µM	Inhibited the proliferation of MIA PaCa‐2 and Huh7 tumor cells; ameliorated vascular smooth muscle cells calcification	Attenuated vascular calcification in CKD mice	Occupied the pocket of the adenosine moiety of SAM	/	[307, 308]
	Lobeline	/	Enhanced the antiproliferative activity of Lenvatinib against HCC cell lines	Reversed lenvatinib resistance in liver cancer xenograft mouse models	Bond to Ile 378, Pro 397, Phe 534, and Asn 549 of METTL3	/	[309]
	Compound C3	/	Inhibited the proliferation of NCI‐H1975 and PC‐9 lung cancer cells	/	Occupied a specific hydrophobic pocket	/	[310]
	F039‐0002 and 7460‐0250	40 and 16.27 µM	/	Ameliorated intestinal inflammation in dextran sodium sulfate‐induced colitis mouse model	Blocked METTL3's catalytic pocket	/	[311]
METTL14	WKYMVM	/	Enhanced the antiproliferative activity of CDK4/6 inhibitors against breast cancer cells	Reversed CDK4/6 inhibitor resistance in breast cancer xenograft mouse models	Occupied METTL3 binding pocket	/	[312]
METTL3‐METTL14 complex	CDIBA‐43n	2.81 µM	Inhibited the proliferation of AML cells	/	Allosteric inhibitor	/	[313]
	Eltrombopag	∼4 µM	Inhibited the proliferation of AML cells	/	Allosteric inhibitor	Yes	[314]
METTL16	Compound 45	1.7 µM	Inhibited the proliferation of MDA‐MB‐231 cells	/	Blocked RNA‐binding site of METTL16	/	[315]
	CDH24	/	Inhibited the proliferation of multiple cancer cells, including AML cell lines, lymphoma cell lines, cervical cancer cell lines, pancreatic cancer cell lines, and GBM cell lines	/	/	/	[316]

**TABLE 2:** Summary of representative small‐molecule inhibitors of m^6^A erasers.

		Effects					
Targets	Small‐molecule inhibitor	Enzymatic activity (IC50)	In vitro	In vivo	Potential mode of action	Selectivity	References
FTO	Rhein	30 µM	Enhanced the antiproliferative activity of tyrosine kinase inhibitors against AML cells and colorectal cancer cells	Inhibited breast tumor growth in 4 T1‐hypodermic breast cancer mouse models; reversed tyrosine kinase inhibitors resistance in AML xenograft mouse models	Occupied the binding sites of m^3^T, 2OG, and Fe2^+^	No	[317, 318, 319, 320]
	Mupirocin	/	Inhibited the proliferation of CRC cells	Impeded tumor growth in CRC xenograft mouse models	Occupied catalytic pocket	/	[321]
	Saikosaponin D	460 nM	Inhibited the proliferation of AML cells	Suppressed tumor growth and metastasis, and prolonged survival in AML xenograft mouse models	Occupied the substrate‐binding site	/	[322]
	MA and MA2	8 µM	Inhibited the proliferation of drug‐resistant lung cells and glioma cells	Suppressed tumor growth and prolonged survival in glioblastoma xenograft mouse models	Partially occupied dm^3^T‐ and Rhein‐binding sites	Yes	[323, 324, 325, 326]
	Diacerein	1.5 µM	/	/	Occupied the substrate‐binding site	/	[327]
	Entacapone	3.5 µM	/	Reduced body weight and lowered fasting blood glucose concentrations in diet‐induced obese mice	Occupied both the cofactor and the substrate binding sites of FTO	/	[328]
	CS1 and CS2	142.6 and 712.8 nM	Inhibited the proliferation of multiple cancer cells, including AML cell lines, glioblastoma cell lines, breast tumor cell lines, and pancreatic cancer cell lines	Reduced leukemia burden and prolonged survival in AML and breast cancer xenograft mouse models	Occupied the catalytic pocket	/	[329]
	MO‐I‐500	8.7 µM	Inhibited survival and colony‐forming ability of SUM149‐Luc cells	Exhibited anticonvulsant activity in the 6 Hz mouse model	2OG‐competitive	/	[330, 331]
	R‐2HG	133.3 µM	Inhibited the proliferation of AML cells and brain tumor cells	Impaired the engraftment and prolonged survival in AML mouse models	2OG‐competitive	No	[332]
	FB23, FB23‐2, Dac85, and Dac590	0.06 µM, 2.6 µM, 0.7 µM, and 6.06 nM	Inhibited proliferation of AML cells and brain tumor cells; alleviated allergic inflammation in IL‐4/IL‐13‐treated epithelial cells; mitigated motor neurons degeneration	Impaired the engraftment and prolonged survival in AML, HCC, melanoma, CRC, and ccRCC mouse models; suppressed allergic inflammation in allergic airway inflammation mouse model; accelerated wound healing in diabetic rats	Occupied the substrate‐binding site	Yes	[333, 334, 335, 336, 337, 338, 339, 340]
	Dac51	0.4 µM	Inhibited the glycolytic capacity of tumor cells	Increased tumor‐infiltrating T cells impaired the engraftment and prolonged survival in melanoma and colon cancer mouse models	Occupied the substrate‐binding site	Yes	[341]
	Fluorescein	3.23 µM	/	/	Occupied the substrate‐binding site	Yes	[342]
	12o/F97	0.45 µM	Inhibited the proliferation of AML cells	Impeded tumor growth in AML xenograft mouse models	Occupied the substrate‐binding site	Yes	[343]
	Compound 8t	7.3 µM	Inhibited the proliferation of AML cells	Impeded tumor growth in AML xenograft mouse models	Substrate/2OG dual competitive	Yes	[344]
	C6	780 nM	Inhibited the proliferation of esophageal cancer cells	Inhibited tumor growth in esophageal cancer xenograft mouse models	Occupied the substrate‐binding site	/	[345]
	FTO‐04 and FTO‐43N	3.39 and 5.5 µM	Inhibited glioblastoma stem cell neurospheres formation; inhibited the proliferation of glioblastoma cells, AML cells, and gastric cancer cells	/	Occupied the substrate‐binding site	Yes	[346, 347]
	Hydrazide‐MA hybrid analogues (11b)	87 nM	Inhibited the proliferation of AML cells	/	Substrate/2OG dual competitive	Yes	[348]
	18077 and 18097	1.43 and 0.64 µM	Inhibited the proliferation of HeLa and MDA‐MB‐231 cells	Suppressed tumor growth and metastasis in breast cancer xenograft mouse models	Occupied the substrate‐binding site	Yes	[349]
	CHTB, N‐CDPCB, Radicicol	39.24, 4.95, and 16.04 µM	/	/	Occupied the substrate‐binding site	/	[350, 351, 352]
	4‐amino‐8‐chloroquinoline‐3‐carboxylic acid	1.46 µM	Promoted the survival of dopamine neurons	/	/	/	[353]
ALKBH5	W23‐1006	3.848 µM	Inhibited TNBC cell proliferation and migration	Suppressed tumor growth and metastasis in breast cancer xenograft mouse models	Covalently bond to the Cys200 located outside of the catalytic pocket	Yes	[354]
	TD19	1.5–3 µM	Inhibited the proliferation of AML and GBM cell lines	/	Covalently bond to the Cys100 or Cys267 located outside of the catalytic pocket	Yes	[23]
	MV1035	/	Inhibited the migration of U87‐MG, A549, and H460 cell lines	/	2OG‐competitive	No	[355]
	Ena15 and Ena21	18.3 and 15.7 µM	Inhibited the proliferation of GBM‐ derived cell lines	/	Occupied the catalytic pocket	Yes	[356]
	DDO‐2728	2.97 µM	Inhibited the proliferation of AML cell lines	Impeded tumor growth in AML xenograft mouse models	Occupied the substrate‐binding pocket	Yes	[357]
	DDO‐02267	2.53 µM	Inhibited the proliferation of AML cell lines	/	Covalently bond to the Lys132‐related substrate recognition	Yes	[358]
	Compound 18	11.8 µM	Inhibited the proliferation of AML cell lines	/	/	Yes	[359]
	(2‐((1‐hydroxy‐2‐oxo‐2‐phenylethyl)thio)acetic acid	0.84 µM	Inhibited the proliferation of AML cell lines	/	Bond to the active site	/	[360, 361]
	ALK‐04	/	Did not affect proliferation of B16 melanoma cells	Enhanced the efficacy of cancer immunotherapy	/	/	[362]
	20m	21 nM	/	/	Occupied the substrate‐binding pocket	Yes	[363]
	ZINC78774792 and ZINC00546946	/	Alleviated hypoxia‐induced cardiac fibroblast pyroptosis	Improved myocardial infarction‐induced heart failure in murine models	Bond to the active site	/	[364]

#### 7.1.1 Small‐Molecule Inhibitors of M^6^A Writers

##### 7.1.1.1 METTL3 Inhibitors

METTL3‐14 is an important m^6^A methyltransferase complex, and the development of inhibitors of METTL3‐14 is of great significance to the treatment of related diseases. As a source of methyl, many inhibitors are based on the S‐adenosylmethionine (SAM) binding pocket, such as STM2457, UZH1a, and EP652 [292, 300]. The METTL3 IC_50_ of STM1760 is approximately 50 µM. After improvement, the METTL3 IC_50_ of STM2457 is 16.9 µM [292]. STM2457 inhibits the progression of various cancers, including liver hepatocellular carcinoma, colorectal cancer, lung adenocarcinoma, gastric cancer, and neuroblastoma tumor [293, 294, 295, 296, 365, 366, 367]. Nanoparticles composed of poly lactic acid‐hydroxyacetic acid were loaded with STM2457 and the cell‐penetrating peptide TAT, and then encapsulated within a tumor cell membrane‐mimetic nanodrug delivery system modified with the YSA peptide. This nanodrug delivery system demonstrated significant inhibition of tumor growth and metastasis in EPH receptor A2 ‐overexpressing gastric cancers. Meanwhile, combinatorial therapy with this nanodelivery platform and anti‐PD1 antibodies exhibited synergistic antitumor effects [296]. Shan et al. developed an STM2457‐conjugated glutathione‐responsive biomimetic nanomedicine. This innovative treatment suppressed tumor growth in tumor‐bearing mice by reversing epithelial–mesenchymal transition‐mediated drug resistance through a METTL3‐m^6^A‐dependent mechanism when administered in combination with chemotherapy. Notably, STM2457‐conjugated glutathione‐responsive nanovesicles enhanced chemotherapy sensitivity of circulating tumor cells derived from breast cancer patients [297]. Furthermore, Li et al. showed that STM2457‐loaded erythrocyte microvesicle‐derived nanodrug delivery system markedly ameliorated cardiac inflammation and fibrosis in a cardiac fibrosis and remodeling model of mice. Mechanistically, STM2457 effectively modulates polarization and migration of monocyte/macrophage by reducing m^6^A modification on *MyD88* and *TGF‐β1* mRNAs. This study provides a potential therapeutic strategy for cardiac remodeling associated with device implantation [298]. Additionally, numerous derivatives similar to STM2457 have been discovered [368, 369, 370, 371, 372, 373]. Exhilaratingly, one of the derivatives, STC‐15, has advanced into Phase I/II clinical trials for the treatment of non–small cell lung cancer, squamous cell carcinoma of the head and neck, melanoma, acute leukemia, and endometrial cancer (NCT05584111, NCT06975293).

In addition, Moroz‐Omori et al. reported that UZH1a, as a small molecule METTL3 inhibitor, can reduce the mRNA m^6^A/A ratio in three different cell lines (MOLM‐13 cells, U2OS cells, and HEK293T cells). Additionally, UZH1a can specifically inhibit m^6^A modification without inhibiting m^1^A, m^7^G, and m^6^Am [302]. X‐ray crystallography analysis showed that UZH1a inhibitors occupy the pocket of the adenosine moiety of SAM, but do not occupy the pocket of the SAM methionine. Subsequently, Dutheuil et al. optimized and designed the compound EP652, which was modeled after UZH1a and STM2457‐like structures bound to the truncated METTL3/14 complex. This compound can efficiently and specifically inhibit the catalytic activity of METTL3/14 enzymes, and IC_50_< 2 nM. The ability of EP652 to reduce m^6^A modification levels is more pronounced than that of STC‐15. In addition, EP652 inhibits tumor cell proliferation and corrects changes in the biomarkers of oncogenic drivers [300]. Furthermore, they have developed a compound named EP102, which has entered Phase I clinical trials for the treatment of advanced or metastatic malignant solid tumors (NCT07163325).

Berberine hydrochloride was discovered to be a potential METTL3 inhibitor through molecular docking‐based virtual screening. Subsequently, enzymatic activity assays revealed that coptisine chloride, an analog of berberine hydrochloride, exhibited high biological activity in inhibiting METTL3. On the one hand, coptisine chloride significantly downregulated the protein levels of BCL‐2, c‐MYC, and PTEN, thus inhibiting the proliferation of MOLM‐13 cells. On the other hand, coptisine chloride suppressed NLRP3 inflammasome activation in periodontal destruction via inhibiting METTL3‐mediated m^6^A modification of the transcript TNFAIP3 [304]. Furthermore, METTL3 inhibitor compound 54 can effectively inhibit MOLM‐13 cell proliferation, which inhibited METTL3 catalytic activity by filling the SAM binding pocket of METTL3, with an IC_50_ of 54 nM. The thermal shift assay demonstrated that compound 54 did not induce shifts for either METTL1 or METTL16 under 200 µM conditions, suggesting that compound 54 exhibits high selectivity for METTL3 [305]. METTL3 protein structure‐based potency was optimized based on its favorable profile after extensive structure‐based design approaches and multiparameter optimization.

Two adenine derivatives, including N‐substituted amide of ribofuranuronic acid analogs of adenosine and adenosine mimics with a six‐member ring instead of ribose, were shown to have good ligand efficiency and inhibitory potency against METTL3 [374]. Following extensive structure‐based design strategies and multiparameter optimization, UZH2 was subsequently identified as a highly potent METTL3 inhibitor due to its favorable properties. Treatment with UZH2 in MOLM‐13 and PC‐3cells can significantly reduce the mRNA m^6^A modification level and inhibit cell proliferation [306]. Taking advantage of the targeted selectivity of UZH2 for the METTL3‐14 complex, UZH2 was linked to proteolysis targeting chimeras (PROTACs) to facilitate the degradation of METTL3‐14, thereby suppressing the proliferation of AML cells and prostate cancer cells [301].

Several flavonoid natural products have been identified as METTL3 inhibitors, including quercetin, luteolin, and baicalin. Their IC_50_ values for inhibiting METTL3 enzymatic activity were 2.73, 6.23, and 19.93 µM, respectively. Studies showed that quercetin exhibited significant antiproliferative efficacy against MIAPaCa‐2 and Huh7 cells by inhibiting METTL3 activity and reducing vascular calcification in a dose‐dependent manner [307]. Besides, quercetin attenuated vascular calcification by decreasing METTL3‐mediated m^6^A modification of the transcript TNFAIP3 [308]. Molecular docking revealed that quercetin occupied the binding pocket of the adenosine moiety in SAM, but does not occupy the binding pocket of the methionine moiety in SAM [307]. In addition, Lobeline, as another natural product, was found to exhibit METTL3 inhibitory activity through cellular thermal shift assay and molecular docking analysis. Lobeline could effectively improve the resistance of lenvatinib in hepatocellular carcinoma by inhibiting *UBE3B* m^6^A modification [309]. These findings highlight Lobeline's potential as a therapeutic agent in targeting METTL3 and overcoming drug resistance in hepatocellular carcinoma.

Moreover, there are several other small‐molecule inhibitors, based on other METTL3 domains, such as a specific hydrophobic pocket and a catalytic pocket, that have been identified. Compound C3 showed significant inhibitory activity on METTL3 by skillfully housing itself in a specific hydrophobic pocket. Compound C3 can effectively inhibit the proliferation of NCI‐H1975 and PC‐9 lung cancer cells, but its inhibitory effect is significantly weaker than that of STM2457 [310]. In addition, through high‐throughput virtual screening models and enzymatic assays, F039‐0002 and 7460‐0250 were identified as inhibitors of the enzymatic activity of the METTL3/METTL14 protein complex, with IC_50_ values of 40 and 16.27 µM, respectively. Surface plasmon resonance sensorgram showed F039‐0002 and 7460‐0250 blocked METTL3's catalytic pocket. In vivo studies showed that F039‐0002 and 7460‐0250 increase the expression of phosphoglycolate phosphatase (PGP) by inhibiting the activity of METTL3, thus suppressing CD4^+^ T helper 1 cell differentiation and relieving intestinal inflammation [311].

##### 7.1.1.2 METTL14 Inhibitors

Many inhibitors of METTL3 have been developed, but the inhibitors of METTL14, the core component of the methylation complex, are rarely reported. Liu et al. discovered and reported the first and only small molecule WKYMVM with METTL14 inhibitory activity by hybrid virtual screening and detection of m^6^A level. WKYMVM was able to dose‐dependently bind to the METTL3 binding pocket in METTL14. However, WKYMVM exhibited no binding affinity for the two truncation mutants of METTL14 obtained by deleting the METTL3 binding domain of METTL14, indicating that WKYMVM reduced m^6^A levels in cells by blocking the formation of the METTL3‐METTL14 complex. Furthermore, the study revealed that WKYMVM reversed the CDK4/6 inhibitor resistance in breast cancer by inhibiting the METTL14‐m^6^A‐E2F1‐axis [312], making it a promising candidate for overcoming drug resistance in breast cancer and providing a novel perspective for the development of METTL14 inhibitors.

##### 7.1.1.3 Allosteric modulators

Allosteric regulation refers to the phenomenon where the binding of an effector molecule to a protein at an allosteric site alters the protein's binding capacity or activity [375]. 4‐[2‐[5‐chloro‐1‐(diphenylmethyl)‐2‐methyl‐1H‐indol‐3‐yl]‐ethoxy] benzoic acid (CDIBA) is the first allosteric inhibitor of METTL3‐14, which exhibited enzymatic inhibitory activity against METTL3‐14 with an IC_50_ value of 17.3 µM. Compound 43n, an optimized compound based on CDIBA, showed stronger enzymatic inhibitory activity of the METTL3‐14 complex (IC_50_ = 2.81 µM). Additionally, Compound 43n decreased m^6^A levels and exhibited antiproliferative activity against AML cell lines [313].

Eltrombopag has been identified as another allosteric inhibitor of METTL3‐14, which inhibits METTL3‐14 activity in a noncompetitive manner. Through a variety of experimental methods, including molecular docking, evaluating the inhibitory activities of eltrombopag in various METTL3‐14 enzyme forms, as well as the inhibitory activities of its derivatives, it has been indirectly supported that eltrombopag can bind to a new allosteric binding pocket on METTL3. Preliminary in vitro experiments have demonstrated that eltrombopag displayed antiproliferative effects in the MOLM‐13 cell line by inhibiting the activity of METTL3‐14 [314]. The research perspective on allosteric modulators exhibits remarkable innovation, surpassing the limitations of targeting orthosteric sites in conventional drug development and offering novel insights for the development of METTL3‐14 inhibitors.

##### 7.1.1.4 METTL16 inhibitors

In addition to inhibitors targeting METTL3 and METTL14, a few inhibitors have also been identified for METTL16, which serves as another methyltransferase enzyme. Liu et al. discovered several aminothiazolone‐derived compounds that exhibit potent inhibitory effects on METTL16. Notably, Compound 45 emerged as the most effective, with an IC_50_ value of 1.7 µM [315]. In addition, Chen et al. reported that CDH24 and its derivatives can bind to METTL16 and inhibit its activity, thereby inhibiting the proliferation of various tumor cells [316], highlighting the potential of CDH24 as a promising candidate for METTL16‐targeted cancer therapy. Taken together, although the current repertoire of METTL16 inhibitors significantly lags behind that of METTL3‐14 inhibitors in terms of diversity, these pivotal discoveries have effectively cleared the path for further research, development, and exploration of METTL16 inhibitors.

#### 7.1.2 Small‐Molecule Inhibitors of M^6^A Erasers

FTO and ALKBH5 stand out as two pivotal m^6^A demethylases, playing critical roles in the regulation of m^6^A modification dynamics. At present, considerable strides have been made in our understanding of the roles played by ALKBH5 and FTO in physiological and pathological processes, including metabolic diseases, neurological and psychiatric disorders, and cancer [162, 183, 192]. Furthermore, a range of inhibitors targeting these enzymes, especially FTO, have been successfully developed.

##### 7.1.2.1 FTO Inhibitors

In 2010, the crystal structure of FTO and the single‐nucleotide 3meT complex was disclosed, providing strong support for the development of FTO inhibitors [376]. Natural products, as the most diverse compound library, provide a crucial resource for the screening of FTO inhibitors. Rhein, one of the main bioactive components of the traditional Chinese medicine rhubarb, holds the distinction of being the first small‐molecule inhibitor of FTO to be identified. Molecular modeling studies have revealed that rhein reversibly binds to the active sites of m^3^T, 2‐oxoglutarate (2OG), and Fe^2+^ on the FTO, leading to competitively suppress the interaction between FTO with 2OG and m^6^A substrate. The mutation of amino acid R316 in FTO, which is important for 2OG binding, indicates that this pocket is an important site for FTO binding to rhein. However, rhein exhibits substantial off‐target effects, as it not only inhibits FTO activity but also suppresses the activity of other demethylases within the ALKB family, including ALKBH2 and ALKBH3 [317, 377]. Rhein treatment significantly retarded breast tumor growth by targeting FTO [318]. Yan et al. reported that rhein can suppress the proliferation of TKI‐resistant leukemia cells with high FTO expression through targeting the FTO‐m^6^A axis in vivo and in vitro [319]. Additionally, rhein has been shown to augment the sensitivity of colorectal cancer cells to TKIs [320]. Mechanically, rhein exerts an anti‐drug‐resistant tumor effect, which may result from targeting FTO‐mediated AKT/mTOR signaling disorder [378]. These findings pave the way for addressing drug resistance in cancer.

Mupirocin, a bioactive compound isolated from Pseudomonas fluorescens [379], was identified as an FTO inhibitor through a series of screening and validation analyses. Mupirocin notably suppressed the growth of colorectal cancer cells in both in vitro and in vivo models by targeting FTO. Mechanically, mupirocin inhibited the expression of GPX4 and SLC7A11 by binding the catalytic domain of FTO, thus triggering ferroptosis and inhibiting cell proliferation [321]. Targeting ferroptosis represents a promising potential therapeutic strategy for cancer treatment [380]. Furthermore, mupirocin synergistically enhances the antitumor effect of Erastin or RSL3. These findings emphasize mupirocin's potential as a promising therapeutic agent for FTO‐targeted colorectal cancer therapy.

Saikosaponin D, a triterpenoid saponin isolated from Bupleuri Radix, exhibits anti‐inflammatory, antibacterial, antitumor, and antiallergic properties [381]. The enzyme activity assay and molecular docking showed that Saikosaponin D could bind to the FTO substrate‐binding site and inhibit its enzyme activity. Preliminary in vitro experiments have demonstrated that Saikosaponin D exerts antileukemia effects through multiple pathways, including inhibition of proliferation, induction of G1 cell cycle arrest, and apoptosis. Consistently, Saikosaponin D not only prolonged survival in mice xenotransplanted with AML primary cells, but also reverses the tyrosine kinase inhibitors resistance and enhances the therapeutic efficacy in AML by targeting FTO/m^6^A‐mediated pathways [322]. These findings provide a rationale for combination therapy strategies, especially for relapsed/refractory AML patients. In general, despite the fact that natural products usually display a multitude of effects, they present a wide variety of structural frameworks conducive to the development of FTO inhibitors

Employing clinical drugs as a screening resource for FTO inhibitors offers a swift and convenient strategy for new drug development. Meclofenamic acid (MA), a nonsteroidal anti‐inflammatory drug, binds to the N‐terminal domain of the α3 helix in FTO at a site that overlaps with the binding position of rhein's moiety. This interaction inhibits FTO's demethylation activity, as determined through fluorescein‐labeled ssDNA substrate and restriction enzyme digestion assays. Furthermore, MA showed significantly higher selectivity for FTO than ALKBH2, ALKBH3, and ALKBH5 [323]. A study has revealed that the dynamic m^6^A methylome possesses the capability to reversibly modulate drug resistance in tumors. MA, functioning as an FTO inhibitor, effectively inhibited the FTO‐mediated augmentation of mRNA stability in proliferation/survival transcripts, consequently enhancing the sensitivity of tumor cells to tyrosine kinase inhibitors [319]. Furthermore, breast cancer resistance protein (BCRP) and multidrug resistance protein 7 (MRP7) are significant contributors to gefitinib resistance in NSCLC [382, 383]. Mechanistically, MA counteracted the gefitinib resistance of NSCLC cells by inhibiting FTO, which led to an elevation in oncogenic MYC m^6^A modification. This, in turn, reduced MYC protein expression, ultimately downregulating the expression of both BCRP and MRP7 [324]. Based on permeability considerations, the ethyloform of MA dramatically suppressed glioblastoma stem cell growth and self‐renewal by targeting the FTO/m^6^A/MYC axis, thus inhibiting cancer progression in glioblastoma xenograft mouse models [325]. Another study demonstrated that MA synergistically enhanced the antiproliferative effects of temozolomide in glioma cells, suggesting its potential as a novel therapeutic strategy for glioma treatment [326].

Diacerein, another nonsteroidal anti‐inflammatory drug, has been identified as a potential FTO inhibitor. Zhang et al. developed a single quantum dot (QD)‐based fluorescence resonance energy transfer (FRET) biosensor to assess the inhibitory effects of potential FTO inhibitors on FTO demethylation activity, and using this approach, they obtained a highly selective FTO inhibitor, diacerein. The absence of a significant effect of 2OG or Fe^2+^ supplementation on diacerein‐mediated inhibition of FTO demethylase activity indicates that diacerein neither mimics 2OG chemically nor chelates Fe^2+^ to inhibit FTO demethylation. In addition, molecular modeling studies showed that diacerein competitively binds to the substrate binding pocket in FTO, thus exerting its inhibitory effect on FTO enzymatic activity [327].

Entacapone is a catechol O‐methyltransferase inhibitor used to treat Parkinson's disease [384]. Structure‐based virtual screening of U.S. Food and Drug Administration‐approved drug and enzyme activity assays demonstrated that entacapone exhibits potent inhibitory activity against FTO, with an IC_50_ value of 3.5 µM. Docking analysis and crystallography revealed that entacapone occupied both the cofactor and the substrate binding sites of FTO. In vivo, entacapone modulated body weight and blood glucose levels by targeting the FTO‐FOXO1 axis in an m^6^A‐dependent manner [328]. Inhibitors derived from previously developed drugs often exhibit off‐target enzymatic activities and lack specificity for FTO, thereby prompting researchers to develop more effective, specific, and selective FTO inhibitors.

CS1 and CS2 are called bisantrene and brequinar, respectively, and they have both advanced to clinical trials for cancer treatment [385, 386]. High‐throughput virtual screening and validation analyses showed that CS1 and CS2 are potent small‐molecule FTO inhibitors. CS1 and CS2 significantly elevated mRNA m^6^A levels in tumor cells, particularly in *MYC*, *CEBPA*, and *LILRB4* transcripts, leading to reduced protein expression and subsequent suppression of cancer stem cell self‐renewal alongside reprogramming of the cancer cell immune response [329]. Currently, CS1 has been approved for clinical trials in the treatment of acute myeloid leukemia [387]. These studies further spurred researchers’ interest in leveraging FTO inhibitors for disease therapy.

Beyond relying on natural products and clinical drugs, identifying novel compound scaffolds is crucial for the development of FTO small‐molecule inhibitors. To date, several FTO inhibitors with distinct chemical skeletons have been reported, including 2,4‐PDCA, IOX1, FB23, and FTO‐02, etc. Although 2,4‐PDCA and IOX1 exhibit potent inhibitory activity against FTO, they also have strong binding ability to other 2OG‐dependent oxygenases, leading to unintended off‐target effects [388]. Therefore, researchers have been motivated to utilize these compounds as lead scaffolds for developing more efficacious inhibitors.

MO‐I‐500, a synthetic ascorbic acid analog, effectively inhibited FTO activity by targeting its 2‐oxoglutarate binding site, with an IC_50_ of 8.7 µM. Notably, the binding site of MO‐I‐500 on FTO closely resembles that of prolyl‐4‐hydroxylase. Treatment with MO‐I‐500 significantly elevated cellular m^6^A levels, comparable to the effects of FTO knockdown. Furthermore, MO‐I‐500 exhibited anticonvulsant activity in the 6 Hz mouse model, positioning its potential as a novel therapeutic candidate for neurological disorders [330]. Additionally, treatment of SUM149 cells with 2 µM MO‐I‐500 significantly suppressed proliferation and colony formation under glutamine‐depleted conditions, whereas no such inhibitory effect was observed in glutamine‐supplemented media [331]. This study offered valuable insights for overcoming therapeutic resistance in TNBC. R‐2‐hydroxyglutarate (R‐2HG), a 2‐oxoglutarate analog produced by mutant isocitrate dehydrogenases (IDHs) via α‐ketoglutarate catalysis, competitively inhibits a broad spectrum of Fe^2+^/α‐KG‐dependent dioxygenases [389]. Su et al. reported that R‐2HG suppressed cancer cell proliferation and promoted cell cycle arrest and apoptosis, which was caused by destabilizing *MYC* and *CEBPA* mRNA through the inhibition of FTO activity (exhibiting an IC_50_ of 133.3 µM). In addition, R‐2HG inhibited FTO transcription through a positive feedback loop driven by CEBPA suppression, thereby further enhancing its antiproliferative effects. However, the inherently low levels of FTO in R‐2HG‐resistant cells render R‐2HG incapable of exerting its anticancer effects [332], suggesting that chronic R‐2HG treatment may induce tumor cell desensitization.

FB23, a derivative of MA, exhibits an IC_50_ value of 0.06 µM for FTO inhibition, demonstrating over 140‐fold greater inhibitory potency compared with MA. In the extended heterocyclic ring of FB23, a hydrogen bond forms between the nitrogen or oxygen atom of FB23 and the amide backbone of Glu234 in FTO. This interaction likely confers FB23 with higher specificity and inhibitory activity compared with MA [333]. Lian et al. uncovered that FB23 relieved allergic inflammatory responses through targeting FTO in vivo and in vitro. Mechanistically, FB23 treatment rectified IL‐4/IL‐13 or house dust mite‐mediated dysregulation of TNF signaling through inhibiting FTO [334]. These studies offer valuable insights for the treatment of asthma. Due to the low cellular uptake of FB23 by NB4 and MONOMAC6 cells, its IC_50_ value for inhibiting cell proliferation exceeds 20 µM. To address this, the FB23 structure was optimized, yielding FB23‐2 with enhanced permeability. FB23‐2 exhibited a more potent inhibitory effect on the proliferation of AML cell lines, with its IC_50_ ranging from 0.8 to 5.2 µM. FB23‐2 significantly suppressed leukemia progression and prolonged survival by targeting oncogenic FTO in mice xenotransplanted with AML cells [333]. In addition, FB23‐2 dramatically inhibited tumor progression and prolonged survival through the FTO‐SIK2‐autophagy axis in the patient‐derived xenograft clear cell renal cell carcinoma mice model [335]. Furthermore, Zhu et al. have developed a nanomedicine that concurrently possesses antigen‐capturing capability and encapsulates FB23‐2, which promotes dendritic cells maturation and immune response through upregulating the m^6^A modification level, thus inhibiting distant tumor growth and metastasis in Hepa1‐6 tumors of mice [336]. These findings highlight the potential of FB23‐2 as a promising candidate for FTO‐targeted cancer therapy. Apart from its anticancer properties, FB23‐2 has the capacity to restore the m^6^A modifications of several risk genes associated with amyotrophic lateral sclerosis, thereby significantly protecting motor neurons from degeneration and ameliorating motor impairments [337]. FB23‐2‐loaded nanocolloidal hydrogel accelerated wound healing in diabetic rats. Mechanistically, FB23‐2‐loaded nanocolloidal hydrogel decreased the *Mmp9* mRNA stability by increasing *Mmp9* m^6^A modification levels, thus elevating MMP9 expression and promoting collagen deposition in wounds [338].

Building upon the development of FB23 and its optimized derivative FB23‐2, Yang's team designed small‐molecule inhibitors Dac51 and Dac85 with potent FTO‐targeting activity. Dac51 suppressed FTO‐mediated immune evasion and synergized with immune checkpoint blockade to prevent tumor recurrence by reversing metabolic reprogramming through m^6^A‐dependent epitranscriptomic enhancement of c‐Jun, JunB, and C/EBPβ expression [341]. Dac85 is derived from FB23 by substituting the chlorine atom at the amino ortho‐position of the benzene ring with a fluorine atom. It exhibits significantly enhanced anti‐AML cell proliferation activity, and its inhibitory effect on FTO enzyme activity is superior to that of FB23‐2 [339]. Additionally, structural optimization of the FB23 scaffold yielded ZLD115, wherein the chlorine atom at the amino ortho‐position of the benzene ring was replaced by a cyclopropyl moiety, and a flexible basic side chain was introduced into the solvent‐exposed cavity. ZLD115 exhibited superior permeability and oral bioavailability compared with Dac85. Moreover, ZLD115 effectively inhibited leukemia progression by upregulating RARA expression and downregulating MYC expression in the xenograft mouse model [390]. Structural optimization of the FB23 scaffold via phenyl bioisosteric substitution and electron‐rich group incorporation generated 12O/F97, a highly selective FTO inhibitor (IC_50_ = 450 nM). Crystallographic studies revealed that 12O/F97 binds FTO in a manner analogous to FB23, with key interactions at the catalytic pocket. Consistent with ZLD115, 12O/F97 exhibited antileukemia effects by upregulating RARA expression and downregulating MYC expression in AML cell lines and xenograft mouse models [343].

In addition, Yang et al. demonstrated that fluorescein and its derivatives exhibit potent FTO enzymatic inhibitory activity. X‐ray crystallographic analysis revealed that their binding site partially overlaps with the MA‐binding region and partially with the nucleotide‐binding site. Furthermore, they reported that fluorescein derivatives can be employed for FTO protein labeling and enrichment [342]. Subsequently, they optimized the structure by integrating features from Dac85 and fluorescein, yielding Dac590, which is a potent, specific, and selective FTO inhibitor, with an IC_50_ value of 6.06 nM. Notably, oral administration of Dac590 effectively suppressed leukemia progression in xenograft mouse models, whereas oral DAC85 lacked such antileukemic activity, suggesting Dac590 has superior bioavailability compared with Dac85 [340]. Most recently, they designed a series of substrate/2OG dual‐targeted inhibitors, drawing on identified FTO inhibitors and the pharmacophore principle. It is worth mentioning that compound 8t exhibits potent FTO inhibitory activity, with an IC_50_ value of 7.3 µM. Compound 8t displayed potent antiproliferative capacities against AML cells and exhibited tumor‐selective accumulation, effectively inhibiting tumor growth in AML xenograft mouse models. At the molecular level, compound 8t suppressed MYC and CEBPA expression while inducing ASB2 and RARA expression via blockade of the FTO‐m^6^A axis, a key oncogenic pathway in AML. However, compound 8t also exerted a strong inhibitory effect on ALKBH3, while showing a relatively weak inhibitory effect on ALKBH5, indicating that compound 8t has certain off‐target effects [344]. Collectively, Yang's group has advanced the field of FTO inhibitor development, and these findings underscore the potential of FTO inhibitors as promising candidates for targeted leukemia therapy and provide robust evidence supporting the development of epitranscriptome‐modifying drugs as a viable therapeutic strategy.

C6, a 1,2,3‐triazole‐pyridine hybrid‐based FTO inhibitor, engages the FTO protein through its pentafluorobenzoyl moieties at a binding site analogous to that of FB23‐2. Notably, C6 exhibited significantly stronger FTO inhibitory activity compared with FB23‐2 [345]. Due to the fact that part of the C6 structure is composed of a nitrogen‐containing heterocycle, it exhibits potent anticancer activity [391]. C6 remarkably suppressed the proliferation of esophageal cancer cells by inducing G2‐phase arrest and significantly reduced tumor growth in oesophageal cancer xenograft models. Mechanistically, C6 suppressed the epithelial–mesenchymal transition pathway and regulated the PI3K/AKT pathway by targeting FTO [345]. In addition, C6 has no obvious toxicity to major organs. These findings underscore the potential of C6 as a promising therapeutic candidate for esophageal cancer therapy targeting FTO.

Based on the MA binding site of FTO, Huff et al. identified a class of pyrimidine‐based FTO inhibitors. These inhibitors exhibit significantly higher selectivity for FTO compared with ALKBH5. It is noteworthy that the IC_50_ values of FTO‐02 and FTO‐04 for FTO inhibition are 2.18 and 3.39 µM, respectively. Furthermore, FTO‐04 inhibited the neurosphere formation of patient‐derived glioblastoma stem cells, but it had no inhibitory effect on the growth of healthy neural stem cell‐derived neurospheres [346]. Based on this study, FTO‐04 was further optimized to target MA binding sites, yielding FTO‐43N. FTO‐43N increased global m^6^A and m^6^Am levels in gastric cancer cells. Its efficacy in inhibiting the proliferation of gastric cancer cells was comparable to that of the clinical chemotherapy drug 5‐fluorouracil or to the efficacy of FTO knockdown, and it exhibited no significant toxicity toward healthy colon cells [347]. While these compounds have exhibited potent antitumor activity in vitro, further in vivo validation is essential to confirm their therapeutic potential.

In addition, Toh et al. identified hydrazide as a potent FTO inhibitor by targeting the 2OG‐ and substrate‐binding sites [392]. Inspired by this study, Prakash et al. developed hydrazide‐MA hybrid analogues targeting both the 2OG‐binding site and substrate‐binding site of FTO through the merging of key structural fragments from known inhibitors. Notably, hybrid analogues (11a and 11b) exhibited significantly stronger inhibitory activity against FTO compared with MA or hydrazide alone. Hydrazide‐MA hybrid analogues elevated intracellular m^6^A levels and suppressed the proliferation of acute monocytic leukemia cells by downregulating *MYC* and upregulating *RARA* mRNA expression [348]. These findings highlight the potential of the hydrazide‐MA hybrid analogue as a promising candidate for clinical treatment in leukemia.

Xie et al. found, through an AutoMD virtual screening approach and restriction endonuclease digestion assay, that AE‐562 and AN‐652 inhibit the demethylation activity of FTO in a concentration‐dependent manner. Subsequently, they analyzed the shared scaffold of AE‐562 and AN‐652, optimized it, and identified compounds 18077 and 18097, which exhibited FTO inhibitory activities by preferentially binding to the substrate‐binding sites of FTO rather than its cofactor‐binding pockets, with IC_50_ values measuring 1.43 and 0.67 µM, respectively. Additionally, the IC_50_ value of compound 18097 against ALKBH5 is nearly 280‐fold higher than that against FTO. Studies revealed that compound 18097 inhibited the demethylation of SOCS1 by targeting FTO and increased the expression of SOCS1 protein in an m^6^A‐IGF2BP1‐dependent manner, resulting in the inhibition of malignant transformation of tumor cells in vitro and in vivo [349]. In addition, Chang's team identified CHTB as a potent FTO inhibitor that significantly increased intracellular m^6^A levels. CHTB binds to the substrate‐binding pocket of FTO, sharing a similar binding mode with meclofenamic acid, but its chemical structure is entirely distinct from that of meclofenamic acid [350]. Previously, they identified a novel FTO small‐molecule inhibitor, N‐CDPCB, which exhibits an IC_50_ of 4.95 µM for FTO inhibition and suppresses FTO‐mediated m^6^A demethylation in cells. N‐CDPCB binds to a specific binding site on FTO, where its cyclobutane ring and phenyl ring engage in hydrophobic interactions with an antiparallel β‐sheet of FTO. Meanwhile, the phenolic ring on the opposite side of the molecule binds to a nonconserved long loop of FTO, anchoring the inhibitor between the antiparallel β‐sheet and the L1 loop. This dual interaction effectively inhibits FTO enzymatic activity [351]. A natural small molecule, Radicol, which shares a binding mode similar to that of N‐CDPCB, was identified as an FTO inhibitor [352]. Radicol, a large cyclic natural product parasitic on the fungus *Monosporium bonorden*, exerts antitumor effects by targeting Hsp90 [393]. These results suggest that the antitumor effect of Radicol may be partly due to its inhibitory effect on the oncogene of FTO.

In addition to the aforementioned small‐molecule inhibitors, the quinolone derivatives 4‐amino‐8‐chloroquinoline‐3‐carboxylic acid and 8‐aminoquinoline‐3‐carboxylic acid were reported to inhibit FTO activity with IC_50_ values of 1.46 and 28.9 µM, respectively. These two FTO inhibitors significantly attenuated neuronal cell death with efficacy comparable to that of glial cell‐derived neurotrophic factor in growth factor deprivation or 6‐OHDA‐induced embryonic midbrain dopamine neuron apoptosis models [353]. These two FTO inhibitors may provide potential therapeutic strategies for FTO‐related neurological diseases. Collectively, small‐molecule inhibitors targeting FTO have undergone extensive development, and some inhibitors have been launched in clinical trials. This advancement is set to significantly expand the range of epitranscriptomic drugs available for treating diseases.

##### 7.1.2.2 ALKBH5 Inhibitors

At present, the development of ALKBH5 inhibitors significantly lags behind that of FTO inhibitors. Similar to FTO, the catalytic pocket of ALKBH5 contains both a 2OG binding domain and a substrate binding domain, suggesting some small molecules can inhibit FTO and ALKBH5. For instance, the IC_50_ value of FTO‐04 for FTO inhibition is 3.39 µM, which differs by merely over tenfold from its IC_50_ for ALKBH5 inhibition (39.4 µM) [346]. In 2014, the crystal structure of ALKBH5 was reported, revealing its substrate recognition region [26]. In addition, Aik et al. uncovered some unique binding sites of ALKBH5 and identified a small molecule inhibitor, IOX3, which can covalently bind to Cys200 located near the active site of ALKBH5 [394]. These studies have paved the way for the development of highly potent, specific, and selective ALKBH5 inhibitors.

ALKBH5 has multiple cysteine residues, including (C100, C227, C230, and C267) around its catalytic domain. W23‐1006, a small‐molecule inhibitor of ALKBH5, was identified through virtual screening and structural optimization. The enzymatic inhibitory effect of W23‐1006 on ALKBH5 is significantly more potent than that on FTO and ALKBH3, which is attributed to W23‐1006 forming a covalent bond with the Cys200 residue located proximal to the catalytic domain of ALKBH5. High ALKBH5 expression is correlated with shorter survival in TNBC patients [395]. With the high efficacy and selectivity for ALKBH5, W23‐1006 significantly suppressed TNBC cell proliferation and metastasis in vitro and in vivo. Mechanistically, W23‐1006 enhances the m^6^A methylation level of fibronectin 1 (FN1) transcripts, thereby leading to degradation of *FN1* mRNA in a YTHDF2‐dependent manner [354]. Among them, FN1 plays a critical role in facilitating the invasion and metastasis of cancer cells [396, 397]. In addition, Lai et al. reported that TD19, an analog of tideglusib, can selectively form covalent bonds with C100 or C267 of ALKBH5, thereby preventing m^6^A substrates from binding to ALKBH5 and increasing intracellular m^6^A levels. TD19 treatment significantly reduced the abundance of *AXL* (AXL receptor tyrosine kinase) and *FOXM1* (Forkhead box M1) mRNA by selectively inhibiting ALKBH5, thereby exerting antiproliferative effects [23]. These findings highlight the potential of ALKBH5 inhibitors to develop residues around the ALKBH5 catalyst pocket for further study as effective cancer inhibitors. These findings highlight the potential of targeting residues surrounding the ALKBH5 catalytic pocket to develop novel ALKBH5 inhibitors for further research on effective cancer therapeutics.

ALKBH5 exhibits high expression levels in glioma stem cells (GSCs) and plays a crucial role in glioma stem cell self‐renewal, proliferation, and tumorigenesis [398]. MV1035, a sodium channel blocker, was found to act as a potent ALKBH5 inhibitor in glioblastoma cells. MV1035 significantly impeded the migration and invasion of U87 glioblastoma cells in a dose‐dependent manner. Mechanically, MV1035 likely suppresses ALKBH5 catalytic activity of ALKBH5 by competing for the binding domain of 2OG, thus elevating the level of m^6^A modification and downregulating CD73 protein expression independent of transcriptional alterations [355]. Furthermore, the inhibitory effects of MV1305 on glioblastoma cell migration and invasion are partially attributable to its suppression of ALKBH2 [399]. Although these results suggest that MV1305 is a potential candidate for targeted therapy of glioblastoma, its potential off‐target effects cannot be overlooked [355].

Additionally, Ena15 and Ena21, potent and selective ALKBH5 inhibitors, were identified by high‐throughput screening and enzymatic activity assays. These compounds likely bind to the catalytic pocket of ALKBH5, thereby suppressing the growth activity of glioblastoma multiforme, with an effect comparable to that of ALKBH5 knockdown. Mechanically, Ena15 and Ena21 inhibit glioblastoma cell proliferation and arrest the cell cycle by enhancing the stability of *FOXM1* mRNA in an ALKBH5‐m^6^A‐dependent manner [356], where FOXM1 plays a critical role in the progression of glioblastoma [398, 400]. These findings offer valuable insights for glioblastoma therapy with the development of ALKBH5‐specific inhibitors.

Compound 8539‐0746 was identified as being more selective for ALKBH5 than for FTO and ALKBH3 through a virtual screening of compounds from the Chemdiv and SPECS databases. Following this, an additional round of structural optimization was performed on Compound 8539‐0746, yielding DDO‐2728, which exhibited markedly enhanced inhibitory activity against ALKBH5. Docking studies and microscale thermophoresis assays revealed that DDO‐2728 does not bind to the 2OG pocket; instead, it binds to the substrate recognition and binding regions. DDO‐2728 exerts antileukemic effects by targeting ALKBH5‐TACC3 signaling axis both in vitro and in vivo [357]. Consistently, ALKBH5 knockdown significantly reduces the mRNA half‐life of the proliferation‐associated oncogene TACC3 by increasing its m^6^A modification, thereby inhibiting AML cell reproliferation and impairing leukemia stem cell self‐renewal [401]. Furthermore, Guo's team identified DDO‐02267 as another potent and selective ALKBH5 inhibitor, based on a pan‐AlkB protein inhibitor MD‐9 and the critical Lys132 residue within the substrate‐selective recognition domain of ALKBH5 [394]. DDO‐02267 inhibited ALKBH5 enzyme activity by covalent binding to Lys132 based on the salicylaldehyde warhead. Treatment with DDO‐02267 significantly increased the levels of cellular m^6^A and inhibited receptor tyrosine kinase AXL expression by targeting ALKBH5, resulting in cell cycle arrest and apoptosis in AML cells [358]. These findings pave the way for ALKBH5‐targeted leukemia therapy.

Furthermore, apart from those mentioned above, other ALKBH5 inhibitors have also been documented, such as ALK‐04, ZINC00546946, and 5‐hydroxy‐1‐(3‐(trifluoromethyl) phenyl)‐1H‐pyrazole‐3‐carboxylic acid (20m). Although ALK‐04 did not directly suppress tumor cell proliferation, it enhanced the inhibition of melanoma tumor growth when combined with GVAX/anti‐PD‐1 therapy. Mechanistically, ALK‐04 increases the stability of the transcript of Mct4/Slc16a3 in an ALKBH5‐m^6^A‐dependent manner, thereby modulating lactate levels and facilitating the recruitment of immune cells in the tumor microenvironment [362]. By targeting ALKBH5, ALK‐04 augments the effectiveness of immunotherapy, underscoring its potential as a supplementary agent to enhance the efficacy of cancer immunotherapy. Fluorescence polarization assays and differential scanning fluorimetry assays showed that 5‐hydroxy‐1‐(3‐(trifluoromethyl) phenyl)‐1H‐pyrazole‐3‐carboxylic acid (20m) selectively binds to and inhibits the enzymatic activity of ALKBH5 by occupying its substrate‐binding pocket, thereby significantly elevating intracellular m^6^A levels in HepG2 cells [363]. In addition, Han et al. identified two potential ALKBH5 inhibitors, ZINC78774792 and ZINC00546946, through virtual screening [402]. In follow‐up studies, these compounds attenuated hypoxia‐induced cardiac fibroblast pyroptosis by inhibiting the Notch1/NLRP3 pathway in an ALKBH5‐m^6^A‐dependent manner [364]. These findings unveil novel therapeutic avenues for targeting cardiovascular diseases.

### 7.2 m^6^A Sites Editing‐Based Therapeutic Strategies

Unlike small‐molecule inhibitors that globally target m^6^A writers (e.g., METTL3/METTL14 complex) or erasers (e.g., FTO, ALKBH5), oligonucleotide‐based strategies have emerged as a powerful and programmable approach to precisely target and manipulate m^6^A modifications. *ALKBH5* mRNA‐loaded exosome‐liposome hybrid nanoparticles significantly suppressed the tumorigenesis of colorectal cancer by reducing m^6^A levels of the *JMJD8* mRNA in vivo [403]. Tang et al. developed a smart delivery system utilizing a pH‐responsive detachable PEG layer and a folic acid‐modified cationic liposome core for co‐delivering doxorubicin and METTL3 siRNA, thereby boosting chemotherapy efficacy by inhibiting m^6^A methylation [404]. This system provides a reliable approach to improving cancer chemotherapy with suppressed chemo‐resistance through rectifying the m^6^A methylome [404]. These studies suggest that oligonucleotide‐mediated targeting of m^6^A‐modifying enzymes offers a novel avenue for cancer therapy.

CRISPR/Cas (Clustered Regularly Interspaced Short Palindromic Repeats) system has been extensively employed for gene editing owing to its high efficiency, ease of use, and accuracy. Liu et al. showed that fusing CRISPR‐Cas9 with METTL3 and METTL14 proteins elevates site‐specific methylation of RNAs. For example, site‐specific methylation of the 5′‐untranslated region (5′UTR) of *Hsp70* promotes mRNA translation [405]. In addition, Cas9 fusion with FTO or ALKBH5 can achieve a reduction in site‐specific methylation of RNAs, such as site‐specific demethylation on A2577 of *Malat1*, resulting in destabilization of the RNA [405]. Ying et al. reported that dRCas9 fused with METTL3 catalytic domain can efficiently increase m^6^A modification in the 3′UTR region of CDCP1 transcripts, thus promoting mRNA translation and bladder cancer development in vitro and in vivo [406]. Compared with the Cas9 system, the Cas13 system exhibits stronger RNA targeting specificity and does not require a protospacer‐adjacent motif [407]. The Cas13b‐METTL3 fusion protein achieved m^6^A editing of *HMBOX1*, revealing that METTL3‐catalyzed methylation on HMBOX1 weakens its transcriptional repression of MDM2, thereby promoting cancer cell malignancy [408]. In addition, Wang et al. demonstrated that dCas13b‐ALKBH5 fusion protein‐mediated site‐specific demethylation of epidermal growth factor receptor (EGFR) and *MYC* mRNA suppresses cancer cell proliferation [409]. Notably, dCasRx, the smallest and most efficient enzyme known within the Cas13 family, exhibits high‐efficiency m^6^A editing capability in vivo and in vitro when fused with methyltransferases/demethylases, potentially providing a therapeutic tool for diseases associated with aberrant m^6^A modifications [410, 411].

Beyond direct m^6^A editing, researchers have developed conditional m^6^A editing strategies, such as optogenetic and chemically inducible approaches. Zhao et al. developed a light‐inducible m^6^A editing system leveraging the optogenetic heterodimer proteins CIBN and CRY2PHR. Upon light stimulation, CIBN undergoes a light‐dependent protein‐protein interaction with CRY2PHR, thereby regulating site‐specific methylation levels [412]. It is worth mentioning that two pairs of light‐inducible heterodimerizing proteins, deta‐phyA/FHY1 and BphP1/PspR2, have been employed for reversible RNA m^6^A editing [413]. This method provides a powerful tool for exploring the relationship between m^6^A modification and its functions. The Targeted RNA m^6^A Erasure (TRME) system, a chemically inducible m^6^A editing strategy, can precisely and reversibly demethylate the targeted m^6^A site of mRNA by doxycycline‐inducible fusion of dCas13a with the ALKBH5 catalytic domain. The TRME system revealed that temporal m^6^A erasure on A1398 of *SOX2* mRNA is sufficient to regulate human embryonic stem cell (hESC) differentiation [414]. Additionally, Liang's team developed two chemically inducible and reversible m^6^A editing tools, leveraging abscisic acid (ABA) and Shield‐1 as regulatory ligands [415, 416].

Beyond engineered enzymes, intrinsic cellular mechanisms also exist where endogenous miRNAs can guide m^6^A modification. For instance, the Wang group revealed that miRNAs can promote m^6^A deposition at their binding sites on target mRNAs through sequence complementarity, uncovering a novel role for miRNAs in regulating m^6^A site‐selectivity and linking this mechanism to cerebellar development and long‐term memory formation [417].

The therapeutic potential of targeting m^6^A is significant, especially in cancer contexts where m^6^A dysregulation is prevalent. While small‐molecule inhibitors for METTL3 or FTO are under exploration, oligonucleotide‐based approaches offer the advantage of precision, potentially reducing off‐target effects. For instance, in the immune landscape of the tumor microenvironment, m^6^A modifications intrinsically influence immune cells like NK cells, macrophages, dendritic cells, and T cells [418]. An oligonucleotide‐based strategy could theoretically be designed to modulate the m^6^A status of key immune‐related transcripts (e.g., STAT5 in NK cells or IRAKM in macrophages) in a cell‐type‐specific manner, thereby reshaping antitumor immunity. Furthermore, the discovery that m^6^A can mark aberrantly processed RNAs (e.g., those from intronic polyA sites or with retained introns) for degradation via pathways like m^6^A‐CDS decay suggests another application: Using antisense oligonucleotides to either protect or target specific transcripts based on their m^6^A status [419]. However, challenges remain, including ensuring efficient in vivo delivery and minimizing unintended immunostimulation.

In summary, oligonucleotide‐based tools represent a versatile and rapidly developing frontier for the precise analysis and functional manipulation of specific m^6^A modifications. These strategies not only deepen our understanding of m^6^A's causal roles in biology but also hold promise for developing novel therapeutics aimed at correcting epitranscriptomic imbalances in disease.

## 8 Conclusion and Future Directions

m^6^A, the most abundant internal chemical modification in eukaryotic mRNA, serves as a central regulator of gene expression. This review systematically outlines the molecular mechanisms of m^6^A modification, compares the advantages and limitations of various m^6^A sequencing technologies, and delineates its roles in both physiological and pathological processes. Furthermore, we summarize the current development of inhibitors targeting m^6^A‐related proteins, providing a theoretical foundation for the precise intervention of m^6^A‐associated diseases. However, critical challenges remain, including gaps in understanding the dynamic regulatory network of m^6^A, its underlying mechanisms in disease progression, and viable pathways for clinical translation.

### 8.1 Current Limitations, Controversies, and Knowledge Gaps

The dynamic regulatory network of m^6^A exhibits considerable complexity, with several critical gaps persisting in our understanding of its precise mechanisms in physiological and pathological processes. Although the deposition, removal, and recognition of m^6^A are known to be mediated by the “writer” (methyltransferase METTL3/METTL14/WTAP complex, METTL16), “eraser” (demethylase FTO/ALKBH5), and “reader” (YTHDF1‐3, IGF2BP1‐3 binding proteins), the synergistic and antagonistic relationships among these regulators remain incompletely elucidated. For example, in cardiac hypertrophy, METTL3 inhibits TFEB‐dependent autophagy via m^6^A modification, whereas ALKBH5 may counteract this effect [420]. However, the spatiotemporal dynamics of their competition within cells require further investigation. In certain diseases, multiple methyltransferases appear to coordinately regulate common outcomes. For instance, the METTL3/METTL14/WTAP complex can promote ferroptosis in cancer cells [421, 422, 423], as can METTL16 [103]. Yet, it remains unclear whether these enzymes act in a temporally coordinated manner, exhibit substrate preferences toward distinct targets converging on the same phenotype, or jointly regulate multiple modification sites on a single transcript to direct alternative metabolic fates. Moreover, high‐resolution structural evidence is still lacking to clarify the molecular basis of their interactions, such as the influence of phosphorylation or ubiquitination on complex assembly. In addition, m^6^A modifications in mRNA are mainly concentrated in the 3′‐untranslated region (3′UTR) and near the stop codon of mRNA, but modification sites in noncoding RNA (such as lncRNA, snRNA, and miRNA) have not been established [424, 425]. For example, some lncRNAs (such as XIST) have m^6^A modifications that regulate their chromatin binding ability, but the molecular logic of why certain lncRNAs are preferentially recognized by writer complexes while other lncRNAs with homologous sequences are not modified remains to be explored. The role of cross‐regulatory networks of m^6^A modifications with other RNA epitranscriptomic modifications (e.g., m^5^C, m^1^A, m^6^Am), and even interactions with epigenetic regulation (e.g., DNA methylation, histone modifications) in disease development has not been clarified, limiting a comprehensive understanding of disease mechanisms.

Second, there is a cognitive blind spot in the synergistic network of m^6^A “reader” proteins. Although the sequential switching mechanism of YTHDC1 and IGF2BP2 in lung progenitor cell differentiation has been revealed [426], the cross‐regulatory effects of other reader proteins (such as the HNRNP family) remain to be systematically studied. Furthermore, the “chicken‐and‐egg” relationship between m^6^A modification and diseases warrants further elucidation. Although m^6^A modifications have been shown to be abnormal in cardiovascular disease, neurodegenerative disease, and cancer, their causal relationship needs to be further verified. For example, in heart failure, m^6^A modifications are involved in disease progression by affecting the translational efficiency of miR‐221‐3p, but there is a lack of gene‐edited animal models to demonstrate the direct pathogenicity of m^6^A modifications [427]. As mentioned above, while a preliminary understanding of the important role of m^6^A in pathophysiology has been established, disease progression is a multistage process, leaving the stage‐specific contributions of m^6^A dynamics largely unexplored. Finally, systematic comparisons of m^6^A conservation across species are limited. Divergent functional outcomes of orthologous modifications in different organisms may undermine the translational relevance of findings from model systems to human clinical contexts.

Although the chemical nature of m^6^A modification is conserved across tissues, its functional outcomes exhibit significant tissue specificity. For example, the same m^6^A chemical modification and FTO regulate metabolic genes in adipose tissue to manage energy, but regulate guidance genes in the nervous system to build neural networks, with completely different functions [192, 285]. The association mechanism between upstream signal pathways (such as hormone signals and metabolite levels), regulated by tissue specificity and m^6^A regulatory factors, still needs to be further explored. Furthermore, functional redundancy among m^6^A reader proteins is increasingly recognized as a common feature in physiological and pathological processes, including embryonic stem cell differentiation and tumorigenesis (such as YTHDF1/2/3, which are involved in translation regulation of gastric cancer) [428]. However, genetic ablation of a single reader protein often results in only partial phenotypic changes, suggesting the existence of robust compensatory mechanisms mediated by parallel readers or yet unidentified regulatory factors.

### 8.2 Emerging Technologies

Since the first transcriptome‐wide m^6^A mapping method using an m^6^A‐specific antibody was reported in 2012, a variety of population cell sequencing approaches have been developed to profile the position of m^6^A sites in the transcriptome, including antibody‐dependent techniques, enzyme‐based approaches, chemically assisted strategies, and direct RNA sequencing. Compared with single‐cell m^6^A sequencing technologies, traditional bulk RNA sequencing techniques are limited in their ability to resolve m^6^A modification dynamics within heterogeneous populations. Emerging single‐cell m^6^A sequencing technologies, including scm^6^A‐seq and sn‐m^6^A‐CT, leverage microfluidic cell sorting, barcoding strategies, and m^6^A‐specific immunoprecipitation to enable rapid and cost‐effective mapping of m^6^A epitranscriptomes at single‐cell resolution. For example, scm^6^A‐seq found differential genomic activation and multiple transcription factor m^6^A modifications among blastomeres in early embryo development, revealing specific regulatory mechanisms for embryo development [71]. However, antibody‐based enrichment approaches such as scm^6^A‐seq are susceptible to m^6^Am co‐enrichment and offer limited single‐nucleotide resolution compared with enzyme‐based strategies like scDART‐seq. The latter system bypasses antibody dependency by employing a fusion protein of cytidine deaminase APOBEC1 and the YTH m^6^A‐binding domain, enabling m^6^A‐dependent C‐to‐U conversion as a proxy for modification sites. A key limitation of scDART‐seq, however, is its reliance on exogenous expression of the engineered APOBEC1‐YTH construct in cells, which restricts its application. All the aforementioned methods require reverse transcription, a process prone to mismatches or deletions. In this regard, nanopore‐based direct RNA sequencing offers a compelling alternative by detecting m^6^A modifications in native RNA strands without cDNA conversion. This technology provides single‐molecule, isoform‐resolved m^6^A profiling with high base‐resolution potential [429]. Nevertheless, nanopore sequencing still requires optimization in several aspects, including mitigating RNA degradation during direct sequencing and refining computational models for accurately calling base‐level modification signals.

Furthermore, while single‐nucleus m^6^A mapping technologies such as sn‐m^6^A‐CT enable spatial transcriptomic profiling of both nuclear and cytoplasmic m^6^A modifications [73], the ability to resolve the dynamic spatial distribution of m^6^A methylomes and transcriptomes in other subcellular compartments remains limited. Beyond the nucleus, epitranscriptomic regulation extends to mitochondria, where modifications such as m^5^C have been shown to mediate metabolic plasticity during tumor metastasis [430]. Resolving the dynamic spatial distribution of m^6^A modifications is particularly critical for understanding functional alterations in specialized cell types, such as neurons [187, 431], multinucleated skeletal muscle cells [432], and tumor cells [433, 434]. Several emerging techniques, including m^6^AISH‐PLA (m^6^A‐specific in situ hybridization mediated proximity ligation assay) [435], m^6^A‐PHPEA (proximity hybridization followed by primer exchange amplification) [436], DART‐FISH (deamination adjacent to RNA modification targets‐ fluorescence in situ hybridization) [437], and the TadA8.20‐assisted m^6^A RNA imaging at single‐base resolution (TARS) [438], now allow quantitative spatial localization of m^6^A‐modified RNAs. Notably, DART‐FISH and TARS can simultaneously achieve quantitative and spatial localization of unmodified transcripts. However, these methods are currently incapable of continuously monitoring the spatial dynamics of m^6^A‐modified transcripts in living cells. Moreover, the application of spatial m^6^A transcriptomics in intact physiological systems, such as the nervous system, requires further methodological development to enable in vivo profiling at cellular resolution.

In addition, the development of targeted m^6^A site editing tools and adenine base editing tools paves novel avenues for precisely understanding the relationship between m^6^A modification and biological functions. Furthermore, the subsequently developed inducible and reversible editing tools targeting specific m^6^A sites provided novel insights for dissecting the dynamic biological function of m^6^A in physiological and pathological processes. For instance, the TRME system regulated embryonic cell differentiation through precise temporal control of site‐specific demethylation of *SOX2* mRNA [414]. While preclinical evidence for targeted m^6^A site editing tools and adenine base editing tools in disease models remains limited, their ability to achieve single‐nucleotide resolution modifications positions them as next‐generation tools for personalized medicine.

### 8.3 Translational Roadmap: From Bench to Bedside

Research on m^6^A RNA modification is rapidly advancing from fundamental discovery to clinical translation, uncovering novel therapeutic targets and strategies for various diseases. The identification of druggable targets, coupled with the development of small‐molecule inhibitors and nucleic acid‐based agents, is laying a solid foundation for m^6^A‐targeted drug development.

In terms of small molecule inhibitors, METTL3 inhibitors (such as STM2457) have been developed, which can significantly inhibit the methyl transfer activity of METTL3 in cancer cell lines, reduce the m^6^A modification level of oncogenes, and induce apoptosis. Notably, STM2457 exhibits significant antitumor efficacy in murine xenograft models. Its derivative, STC‐15, has progressed as the first RNA‐modifying enzyme inhibitor to enter clinical trials. It is currently being evaluated for several oncology indications, including non–small cell lung cancer, head and neck squamous cell carcinoma, melanoma, and endometrial cancer, representing a major milestone for m^6^A‐targeted therapy (NCT06975293).

Regarding nucleic acid‐based inhibitors, combining the RNA targeting capabilities of CRISPR‐Cas13 with an m^6^A‐generating RNA methyltransferase enables targeting of RNA methylation and programmable manipulation of the epitranscriptome [439]. Future advancements in this field will depend on optimizing drug delivery systems. The development of tumor‐targeting antibody–drug conjugates or nanocarriers capable of crossing the blood–brain barrier, for instance, could significantly improve the tissue specificity and bioavailability of these therapeutic agents.

It is worth noting that the combined application of m^6^A‐targeted drugs with traditional therapies or immunotherapy has the potential to provide new therapeutic strategies for drug‐resistant diseases and become an adjunctive therapy for tumor immunotherapy. Furthermore, dynamic alterations in m^6^A RNA methylation exhibit disease‐specific temporal patterns during pathogenesis, conferring potential as biomarkers for noninvasive diagnosis and efficacy monitoring of diseases. The advancement of translational medicine relies critically on seamless and robust collaboration between basic scientists, clinicians, and bioinformatics specialists. For example, RMVar [440], m6A‐Atlas [441], M6AREG [442], and M6A2target [443] have significantly promoted the integration and sharing of global data. Meanwhile, the application of artificial intelligence and deep learning models makes it possible to predict the impact of m^6^A modification on gene expression, providing a tool to support the formulation of individualized treatment plans. In the field of regenerative medicine, m^6^A regulation technology also shows broad prospects. By interfering with the m^6^A modification status of specific genes, stem cells can be effectively guided to differentiate into specific lineages, thereby providing new strategies for organ damage repair and tissue engineering.

However, the clinical translation of m^6^A research still faces many challenges. Firstly, m^6^A detection technology has not yet been unified, and the consistency of results between different platforms, such as MeRIP‐seq and nanopore direct sequencing, is low, limiting the comparability of data and standardization of clinical applications. Secondly, the m^6^A regulatory network is highly complex, and the same regulatory factor, such as METTL3, may play opposite roles in different tumors. Therefore, precise target selection must be combined with disease molecular classification. At the same time, because the effect of single‐target intervention is limited, multitarget joint regulatory strategies need to be explored in the future. In addition, m^6^A regulatory proteins also perform important physiological functions in normal tissues, and systemic administration may cause off‐target toxicity. For example, structural similarities between the catalytic pockets of FTO and human dihydroorotate dehydrogenase enabled FTO inhibitor FB23‐2 to inhibit both enzymes [444]. The importance of off‐target effects on their structurally similar enzymes to optimize their therapeutic potential and minimize unintended consequences should be considered in the development of m^6^A‐related inhibitors such as FTO. Finally, there are significant differences in m^6^A modification profiles between preclinical models and human tissues, underscoring an urgent need to establish organoids or humanized models with more predictive value to enhance the reliability of preclinical evaluation.

## Author Contributions

Linghuan Li and Hanbing Li: Conceptualization, supervision, and funding acquisition. Linghuan Li, Yuanhai Sun, Wanfang Zheng, Lingqin Li, Yaqian Feng, Minyou Qi: Visualization. Linghuan Li, Yuanhai Sun: Writing – original draft. Linghuan Li, Hanbing Li, and Yuanhai Sun: Writing – review and editing. All authors have read and approved the final manuscript.

## Ethics Statement

The authors have nothing to report.

## Conflicts of Interest

The authors declare no conflicts of interest.

## Data Availability

All data generated and/or analyzed during the current study are included in this published article.
